# 2-aminoimidazoles potentiate ß-lactam antimicrobial activity against *Mycobacterium tuberculosis* by reducing ß-lactamase secretion and increasing cell envelope permeability

**DOI:** 10.1371/journal.pone.0180925

**Published:** 2017-07-27

**Authors:** Albert B. Jeon, Andrés Obregón-Henao, David F. Ackart, Brendan K. Podell, Juan M. Belardinelli, Mary Jackson, Tuan V. Nguyen, Meghan S. Blackledge, Roberta J. Melander, Christian Melander, Benjamin K. Johnson, Robert B. Abramovitch, Randall J. Basaraba

**Affiliations:** 1 Mycobacteria Research Laboratories, Department of Microbiology, Immunology, and Pathology, Colorado State University, Fort Collins, Colorado, United States of America; 2 Department of Chemistry, North Carolina State University, Raleigh, North Carolina, United States of America; 3 Department of Chemistry, High Point University, High Point, North Carolina, United States of America; 4 Department of Microbiology and Molecular Genetics, Michigan State University, East Lansing, Michigan, United States of America; Institut de Pharmacologie et de Biologie Structurale, FRANCE

## Abstract

There is an urgent need to develop new drug treatment strategies to control the global spread of drug-sensitive and multidrug-resistant *Mycobacterium tuberculosis* (*M*. *tuberculosis*). The ß-lactam class of antibiotics is among the safest and most widely prescribed antibiotics, but they are not effective against *M*. *tuberculosis* due to intrinsic resistance. This study shows that 2-aminoimidazole (2-AI)-based small molecules potentiate ß-lactam antibiotics against *M*. *tuberculosis*. Active 2-AI compounds significantly reduced the minimal inhibitory and bactericidal concentrations of ß-lactams by increasing *M*. *tuberculosis* cell envelope permeability and decreasing protein secretion including ß-lactamase. Metabolic labeling and transcriptional profiling experiments revealed that 2-AI compounds impair mycolic acid biosynthesis, export and linkage to the mycobacterial envelope, counteracting an important defense mechanism reducing permeability to external agents. Additionally, other important constituents of the *M*. *tuberculosis* outer membrane including sulfolipid-1 and polyacyltrehalose were also less abundant in 2-AI treated bacilli. As a consequence of 2-AI treatment, *M*. *tuberculosis* displayed increased sensitivity to SDS, increased permeability to nucleic acid staining dyes, and rapid binding of cell wall targeting antibiotics. Transcriptional profiling analysis further confirmed that 2-AI induces transcriptional regulators associated with cell envelope stress. 2-AI based small molecules potentiate the antimicrobial activity of ß-lactams by a mechanism that is distinct from specific inhibitors of ß-lactamase activity and therefore may have value as an adjunctive anti-TB treatment.

## Introduction

The ongoing, global spread of tuberculosis (TB), is due in part to the lack of new and more effective antimicrobial drugs to treat drug-sensitive and multidrug-resistant (MDR) strains of *Mycobacterium tuberculosis* (*M*. *tuberculosis*) [1]. In 2015 alone, an estimated 10.4 million people developed TB resulting in 1.4 million deaths. Moreover, the incidence of new MDR-TB cases continues to increase and was estimated at 480,000 [2]. Treating drug-sensitive TB is challenging and requires a minimum six month course of combination antimicrobial drug therapy consisting of the first-line drugs isoniazid, rifampicin, ethambutol, and pyrazinamide that result in undesirable side-effects in some patients [3]. Moreover, treatment of MDR-TB is considerably more difficult and expensive requiring stronger and potentially more toxic drug combination therapy lasting approximately 2 years [2]. In short, the difficulty in controlling TB partially stems from the prolonged treatment using classical chemotherapy and the low treatment success rate in patients infected with MDR strains of *M*. *tuberculosis*. Unfortunately, the current pipeline of new anti-TB drugs for the treatment of resistant infections have unproven efficacy with undesirable side effects [4, 5].

The 2-aminoimidazole (2-AI) class of small molecules are derived from the marine sponge metabolites oroidin and bromoageliferin [6]. Importantly, treatment with 2-AI reversed isoniazid tolerance of attached *M*. *tuberculosis* communities and significantly reduced numbers of viable bacilli when combined with isoniazid in an *in vitro* model of non-replicating persistence [7, 8]. These observations suggested that combining 2-AI compounds and conventional antibiotics can be a viable option to overcome *M*. *tuberculosis* drug-tolerance or resistance as shown for other clinically important Gram-positive and Gram-negative bacteria [6, 9, 10]. For example, 2-AI derivatives were shown to revert oxacillin resistance in methicillin resistant *Staphylococcus aureus* (MRSA) [11], and suppress PmrAB mediated colistin resistance of drug-resistant *Acinetobacter (A*.*) baumannii* [12–14]. Altogether, these reports suggest that 2-AI compounds have promising potential as an adjunctive therapy when combined with antibiotics to treat drug-tolerant or–resistant bacteria.

Since their introduction, ß-lactam antibiotics have proven to be safe and effective at controlling a variety of bacterial infections [15, 16]. However, ß-lactams are not currently used to treat TB, due to the intrinsic resistance exhibited by *M*. *tuberculosis*. The inherent resistance of *M*. *tuberculosis* to ß-lactams is mainly attributed to two mechanisms: a) inactivation of the antibiotics by *blaC* encoded ß-lactamase and b) low permeability of the mycobacteria cell envelope limiting the diffusion of antibiotics such as ß-lactams [16–23]. As occurs in Gram-negative bacteria, mycobacteria have an outer cell membrane [24–26], a major permeability barrier against ß-lactams targeting penicillin binding proteins (PBPs) that reside in the periplasmic compartment [27]. The inner leaflet of the mycobacterial outer membrane is composed of mycolic acids, long fatty acids approximately 90 carbons in length, that are covalently bound to arabinogalactan and tightly packed together effectively blocking the diffusion of hydrophilic molecules. The outer leaflet of the *M*. *tuberculosis* outer membrane is enriched with non-covalently bound lipids such as trehalose dimycolate (TDM) and phthiocerol dimycoserosates (PDIMs) [24, 28]. Together, this outer membrane serves as a low fluidity and low permeability barrier to antibiotics. Since ß-lactams are currently only considered in the treatment of drug-resistant TB, any strategy that circumvents *M*. *tuberculosis* ß-lactam resistance may provide new opportunities to utilize this class of drugs to treat both drug susceptible and drug-resistant strains of *M*. *tuberculosis* [29, 30]. Indeed, there is renewed interest in repurposing ß-lactams to treat TB in combination with ß-lactamase inhibitors [22, 31, 32], which are supported by recent human clinical trials [33].

This study investigated the use of 2-AI compounds to potentiate ß-lactams against *M*. *tuberculosis*, and the mechanisms by which these compounds work. It was hypothesized that 2-AI compounds would interfere with mechanisms conferring *M*. *tuberculosis* intrinsic ß-lactam resistance. Herein it is reported that 2-AI compounds lower MIC values and improve the bactericidal activity of ß-lactams against *M*. *tuberculosis*. 2-AI compounds reduce *M*. *tuberculosis* ß-lactamase activity by altering secretion of the enzyme rather than by directly inhibiting the enzymatic activity as in the case of the classic ß-lactamase inhibitor clavulanic acid. Mechanistic studies revealed that 2-AI treatment alters *M*. *tuberculosis* cell envelope composition, leading to increased permeability and thus increased binding of cell wall targeting antibiotics. Taken together, these data demonstrate that 2-AI compounds potentiate ß-lactam antibiotics through a novel mechanism, which may be further exploited in the development of adjunctive anti-TB therapy against drug sensitive and drug resistant *M*. *tuberculosis*.

## Materials and methods

### Bacterial strains, media and culture conditions

Stock cultures of *M*. *tuberculosis* H37Rv ATCC 27294 and *M*. *smegmatis* mc^2^155 were stored frozen at -80°C in Proskauer-Beck media (50% v/v glycerol) and glycerol stock media (50% v/v glycerol, 7H9, ADC, Tween 80), respectively. For propagation of initial culture, frozen stocks were thawed and sub-cultured in Middlebrook 7H9 media with OADC (0.005% oleic acid, 0.5% bovine serum albumin fraction V, 0.2% dextrose, 0.0003% catalase), 0.2% glycerol, and 0.05% Tween 80 until reaching a desirable optical density (OD) for each experiment. 7H9 and OADC were purchased from BD (Franklin Lakes, NJ, USA). Glycerol and Tween 80 were purchased from Sigma-Aldrich (St. Louis, MO, USA). All experiments using virulent *M*. *tuberculosis* H37Rv were done in a BSL3 laboratory located at Colorado State University. For experiments using the BSL2 strain, *M*. *tuberculosis* H37Rv mc^2^ 6206, bacteria was grown in 7H9 media supplemented with OADC, 0.2% casamino acid (BD, USA), 0.05% tyloxapol, 0.005% L-leucine, 0.0048% D-pantothenic acid, and 0.0025% kanamycin (Sigma-Aldrich, USA). *M*. *tuberculosis* H37Rv mc^2^ 6206 was a kind gift from Dr. William R. Jacobs Jr. at Albert Einstein College of Medicine [34].

### 2-AI compounds

Structures and synthesis of 2B8 (compound 2 in reference [7]) and RA11 were previously disclosed (S1 Fig). Compounds were dissolved at 100 mM as their HCl salts in molecular biology grade DMSO (Sigma-Aldrich, USA) and stored at -80°C until use.

### Broth microdilution method for MIC determination for ß-lactams with or without 2-AI compounds

Determination of ß-lactam MICs against mycobacteria was carried out by using a broth microdilution method with alamarBlue^®^ (Invitrogen, Carlsbad, CA, USA) as previously described [35]. Briefly, in 96-well flat-bottomed cell culture plates (Thermo-Fisher, Waltham, MA, USA), ß-lactams were serially two-fold diluted in 7H9 media starting from the following concentrations: 1024 mg/L (ampicillin, oxacillin, carbenicillin, penicillin V and amoxicillin), 512 mg/L (cefotaxime and ceftazidime), 256 mg/L (cefoxitin), and 16 mg/L (meropenem). *M*. *smegmatis* and *M*. *tuberculosis* H37Rv grown in 7H9 media to an OD_600_ of 0.4 to 0.6 were further diluted 1:20 and inoculated to wells containing ß-lactams. Final volume of each well was 200 μL. Plates were incubated under stationary conditions at 37°C. After 48 h (for *M*. *smegmatis*) or 5 days (for *M*. *tuberculosis*), 20 μL alamarBlue^®^ was added to each well and incubated for an additional 6 h (for *M*. *smegmatis*) or 24 to 48 h (for *M*. *tuberculosis*). MIC was determined as the lowest drug concentration that prevented color change from blue to purple or pink. This was confirmed as MIC_95_ when measured by fluorescence [36]. Briefly, fluorescence was recorded at 560_ex_/590_em_ and % inhibition of reduction was calculated as follows: MIC = 1–100 × (Sample fluorescence intensity–Negative control fluorescence intensity) / (Positive control fluorescence intensity–Negative control fluorescence intensity), where the positive control is *M*. *tuberculosis* without drugs and the negative control is media. All ß-lactams were purchased from Sigma-Aldrich except for carbenicillin and meropenem (Gold Biotechnology, St. Louis, MO, USA).

For MIC determination when ß-lactams are combined with 2-AI compounds, broth microdilution MIC assay was performed in the presence of two-fold diluted RA11 or 2B8 ranging between 7.8125 and 250 μM. One column of the plate contained only 2-AI compounds without any ß-lactams to determine the MIC of 2-AI compounds against mycobacteria. MICs were determined as above and MIC of ß-lactams alone was divided by MIC of ß-lactams combined with 2-AI compounds to calculate fold-reduction of MIC resulting from 2-AI treatment.

### Evaluation of bactericidal activity of ß-lactams against *M*. *tuberculosis*

*M*. *tuberculosis* H37Rv was incubated at 37°C for five days in 96-well flat-bottomed cell culture plates containing different concentrations of ß-lactams alone, or with 2B8 or potassium clavulanate (Sigma-Aldrich, USA). 2B8 was added at 31.25, 62.5, and 125 μM and clavulanate was added at 8 mg/L. Tested concentrations for each ß-lactam were as follows: carbenicillin (2 and 32 mg/L), amoxicillin (2 and 32 mg/L), ceftazidime (1 and 16 mg/L), and meropenem (0.03125 and 0.5 mg/L). After 5 days of incubation, cultures were serially diluted in sterile PBS and plated on Middlebrook 7H11 agar (BD, USA) with glycerol, OADC and 8 mg/L cyclohexamide (Gold biotechnology, USA). The number of CFUs was determined by counting visible colonies after three or four weeks of incubation at 37°C. Bactericidal activity (%) was calculated as follows: 100–100 × (Treatment group CFUs/Control CFUs). For this calculation, control CFUs were obtained from non-treated (for ß-lactams only group), 2B8 only (for 2B8/ß-lactams combination group), and clavulanate only (for clavulanate/ß-lactams combination group) treated samples.

### Collection of *M*. *tuberculosis* culture filtrate protein (CFP)

To obtain *M*. *tuberculosis* H37Rv CFP, it was grown to an OD_600_ of 0.4 to 0.6 in glycerol-alanine-salts (GAS) media containing 0.03% Bacto Casitone (Difco, Franklin Lakes, NJ, USA), 0.005% ferric ammonium citrate (Sigma-Aldrich, USA), 0.4% dibasic potassium phosphate, 0.2% citric acid, 0.1% L-alanine, 0.12% magnesium chloride hexahydrate, 0.06% potassium sulfate, 0.2% ammonium chloride, 0.18% sodium hydroxide, and 1% glycerol (all purchased from Sigma-Aldrich, USA). Subsequently, cultures were centrifuged (×1,700g) for 10 min and supernatants were harvested. Collected supernatant was filtered through a 0.45 μM syringe filter (Millipore, USA) to obtain CFP. Total protein concentration present in CFP was determined using the BCA assay (Pierce, Waltham, MA, USA) following the manufacturer’s instruction. CFP was also obtained from 2-AI compounds treated *M*. *tuberculosis*. Briefly, after the initial culture in GAS media, cells were washed twice with sterile PBS and reconstituted in GAS media to an OD_600_ of 0.4. Cultures were treated with 2-AI compounds (RA11 or 2B8) or clavulanate (8 mg/L) and incubated at 37°C for 24 h and CFP was harvested as described above.

### ß-lactamase activity assay

ß-lactamase activity was evaluated with the colorimetric kit from Biovision (Milpitas, CA, USA). Briefly, a total of 50 μL samples were transferred to a 96-well cell flat-bottomed culture plate and nitrocefin included in the kit was added to a final concentration of 20 μM. Immediately following the addition of nitrocefin to samples, absorbance at 490 nm was monitored every five min for 2 h at 37°C using a Synergy 2 multi-mode plate reader (BioTek, Winooski, VT, USA) to obtain a nitrocefin hydrolysis curve. A standard curve was derived from known amounts of hydrolyzed nitrocefin provided in the assay kit. Total nitrocefin hydrolyzed (nM) per min was calculated from a standard curve. Data were normalized to CFUs or nM nitrocefin hydrolyzed/min/mg of protein.

### Preparation and analysis of *M*. *tuberculosis* cell envelope lipids

*M*. *tuberculosis* H37Rv mc^2^ 6206 BSL2 strain was grown to an OD_600_ of 0.4. Cultures were treated with 2-AI compounds (RA11 or 2B8) at 2.5, 12.5 and 62.5 μM for 24 h in the presence of 0.5 μCi of [1.2-^14^C] acetic acid (113 Ci/mol) and [1-^14^C] propionate (56.7 Ci/mol, MP Biomedicals, Santa Ana, CA, USA). Extractions and preparation of total lipids and cell-bound mycolic acids followed earlier procedures [37]. Total radioactivity counts were measured for all samples prior to thin layer chromatography (TLC) analysis on a silica gel 60-precoated plate F254 (Merck, Kenilworth, NJ, USA) in various solvent systems. For visualization of radiolabeled lipids, silica gel plates were scanned with Typhoon Trio Imager (GE Healthcare, Little Chalfront, UK) for autoradiogram. Densitometry analysis was performed using ImageQuant TL 8.1 (GE Healthcare, UK).

### SDS sensitivity assay

*M*. *tuberculosis* H37Rv was grown to an OD_600_ of 0.4 to 0.6 in 7H9 media with OADC supplement and 0.05% Tween 80, then treated with 125 μM 2B8 for 24 h. After treatment, bacterial pellets were washed twice with sterile PBS and reconstituted with PBS to an OD_600_ of 0.1. Sodium dodecyl sulfate (SDS, Cayman Chemical, Ann Arbor, MI, USA) was added to the cultures to achieve a final concentration of 0.005 and 0.05%. Cultures were plated on 7H11 agar plates at 0, 1, 2, 3 and 4 h post addition of SDS. After three or four weeks of incubation at 37°C, CFUs were enumerated and percent survival through 4 h was calculated for each sample and compared to starting CFUs at 0 h time-point.

### Dye accumulation assays

*M*. *tuberculosis* H37Rv was grown to an OD_600_ of 0.4 to 0.6 in 7H9 media supplemented with OADC and 0.05% Tween 80 and used for an ethidium bromide (EtBr) or Sytox Orange uptake assay as previously described [38, 39]. Briefly, cultures were dispensed in opaque-walled 96-well flat-bottomed cell culture plates (Corning, Corning, NY, USA) and pre-energized with 0.4% glucose (Sigma-Aldrich, USA) for five min. Thereafter, 2-AI compounds (RA11 or 2B8, 125 μM), reserpine (100 mg/L) or thioridazine (80 μM) (Sigma-Aldrich, USA) were added, followed by EtBr (Sigma-Aldrich, USA) and Sytox Orange (Invitrogen, USA) to a final concentration of 10 μM and 100 nM, respectively. For both fluorescent dyes, relative fluorescence units (RFUs) were monitored every two minutes by 530_ex_ nm/590_em_ nm filter using Synergy 2 multi-mode plate reader for 90 min.

### Evaluation of cell envelope permeability and cell membrane integrity using BOCILLIN^®^, BODIPY^®^ FL vancomycin and propidium iodide

*M*. *tuberculosis* H37Rv was grown to an OD_600_ of 0.4 to 0.6 in 7H9 media supplemented with OADC and 0.05% Tween 80, then diluted to OD_600_ of 0.1 in the same media prior to use. Diluted cultures were treated with 62.5 and 125 μM 2-AI compounds (RA11 or 2B8), SDS 0.05% or 20 μM meropenem in combination with 100 μM clavulanate (MCA) [40]. After treating for 30 min, 120 min, or 24 h at 37°C while shaking, cultures were aliquoted into 5 mL polystyrene tubes and stained with 1 mg/L BODIPY^®^ FL vancomycin, 10 mg/L BODIPY^®^-tagged penicillin V (BOCILLIN^®)^) or 15 μM propidium iodide (PI) (Life Technologies, Carlsbad, CA, USA), for 30 min at 37°C in the dark. Cells were pelleted by centrifuging (×1,700g) for 5 min to remove remaining free dye and washed with sterile PBS two times (PI stained samples were not washed, but directly fixed as described below after removing the supernatant). After these washes, PBS was removed and cell pellets were fixed with 4% paraformaldehyde (VWR, Radnor, PA, USA) in PBS for 15 min. After fixation, bacterial cells were analyzed by flow cytometry using an LSRII flow cytometer (BD, USA). The cytometer was adjusted as follows: Forward scatter (FSC) and side scatter (SSC) were set to logarithmic scale, threshold was set at 2000 FSC and SSC, acquisition was set to low (<1000 events/sec) and 10,000 to 50,000 events were collected for each sample. Fluorescence of BODIPY^®^-labeled antibiotics and PI was excited with the 488 nm blue laser and emission detected with the 530/30 nm and 610LP filters, respectively. Data were analyzed using Kaluza 1.3 software (Beckman Coulter, Brea, CA, USA). For competitive inhibition of BODIPY^®^ FL vancomycin binding to *M*. *tuberculosis*, unlabeled vancomycin (Gold Biotechnology, USA) at 50×, 100×, and 500× the amount of BODIPY^®^ FL vancomycin (1 mg/L), was added to 2B8 treated samples prior to the addition of BODIPY^®^ FL vancomycin. Fixed BODIPY^®^ FL vancomycin stained bacteria were also analyzed under a microscope equipped with X-cite 120 fluorescence illuminator (Excelitas Technologies, Waltham, MA, USA).

### RNA isolation and next generation sequencing

For RNA isolation, *M*. *tuberculosis* H37Rv was cultured in 7H9 media to an OD_600_ of 0.4 then treated with 125 μM 2B8 for 2 and 24 h. After treatment, mycobacterial RNA was extracted using trizol/chloroform (Sigma-Aldrich, USA) as previously described with minor modifications [41]. Briefly, cells were resuspended in trizol and disrupted using Zirconia beads (Biospec, Bartlesville, OK, USA) by beating six times for 30 sec and cooling on ice for one min in between. Beads were removed and chloroform was added to trizol (0.2:1, v/v). Samples were vortexed and centrifuged to extract solubilized RNA in the aqueous phase. To precipitate RNA, molecular grade 100% ethanol (Sigma-Aldrich Aldrich, USA) was added to the aqueous phase and incubated at -80°C overnight. The RNA was pelleted by centrifugation (×17,000g) at 4°C, for 15 min followed by washing with 75% ethanol. DNA contamination was removed by treating with 10 μL DNase1 (New England Biomed, Ipswich, MA, USA) at 37°C for 30 min. Then 100 μL of acid phenol (Sigma-Aldrich, USA) was added to the samples and vortexed. After centrifugation (×17,000g) for one min, the aqueous phase was collected and transferred to clean RNAse-free tubes, followed by the addition of 33 μL sodium acetate (Sigma-Aldrich, USA) and 250 μL of 100% ethanol. Samples were gently mixed by inverting and placed at -80°C overnight. Samples were centrifuged (×17,000g) for 15 min at 4°C to collect RNA pellets. Pellets were further washed with 80% ethanol. After ethanol removal, pellets were air-dried at room temperature for five min and reconstituted in 15 μL of RNAse-free water (Corning, USA). Isolated total RNA samples were prepared for next generation sequencing (NGS) using the Illumina Stranded Total RNA Library Prep Kit (Illumina, San Diego, CA, USA) with the Epicentre Ribo-Zero Gram positive bacteria ribosomal RNA depletion (Illumina, USA). After validation and quantitation, the libraries were pooled for multiplexed sequencing. The pool was loaded on one lane of a HiSeq Rapid Run flow cell (v1) and sequenced in a 1×50 bp single end (SE50) format using Rapid SBS reagents. Base calling was performed by Illumina Real Time Analysis (RTA, Illumina, USA) v1.18.61 and output of RTA was de-multiplexed and converted to FastQ format with Illumina Bcl2fastq v1.8.4 (Illumina, USA). To compensate for the low number of reads obtained for one sample, MiSeq (SE50) sequencing was performed with this one library. Reads from both the HiSeq and MiSeq runs were combined for that particular sample. Sequencing data were analyzed following methods similar to those previously described [42]. Briefly, raw reads were subjected to trimming of low-quality bases and removal of adapter sequences using Trimmomatic (v0.32) [43] with a 4 bp sliding window, cutting when the read quality was below 15 (using the Phred33 quality scoring system) or read length was less than 36 bp. Trimmed reads were then aligned to the *M*. *tuberculosis* H37Rv genome (assembly 19595v2) using Bowtie (v1.0.0) [44] with the–S option to produce SAM files as output. Alignment quality control was performed using the HTSeq-qa function within the HTSeq software package (v.0.6.1) [45]. Further graphical quality control analyses were performed using the Qualimap software suite (v2.0) [46]. Sequencing depth was calculated using SAMtools (v1.2) [47]. Aligned reads were then counted per gene feature in the *M*. *tuberculosis* H37Rv genome using the HTSeq software suite (v0.6.1). Differential gene expression was calculated by normalizing the data utilizing the trimmed mean of M-values normalization method [48] and filtering out genes that had <23 counts per million (CPM) within the edgeR package (v3.0.8) in R (v2.15.3) [49]. The transcriptional profiling data have been submitted to the NCBI GEO database (accession no. GSE95773).

### Statistical analysis

Statistical analyses were carried out using one-way ANOVA with Tukey’s post hoc test using GraphPad Prism 5 (GraphPad Software, La Jolla, CA, USA). P values less than 0.05 were considered significant. For RNA transcriptome data, statistical analysis was performed in R Studio (ver. 0.98.1091) by the exact test with a negative binomial distribution for each set of conditions and testing for differential gene expression [50] using edgeR (v3.0.8). Differentially expressed genes were determined to be statistically significant based on a q < 0.05 and >1.5-fold differentially regulated. Magnitude amplitude (MA) plots were generated by modifying a function within the edgeR package (v3.0.8).

## Results

### 2B8 treated *M*. *tuberculosis* fails to grow in the presence of carbenicillin

**Fig 1:**
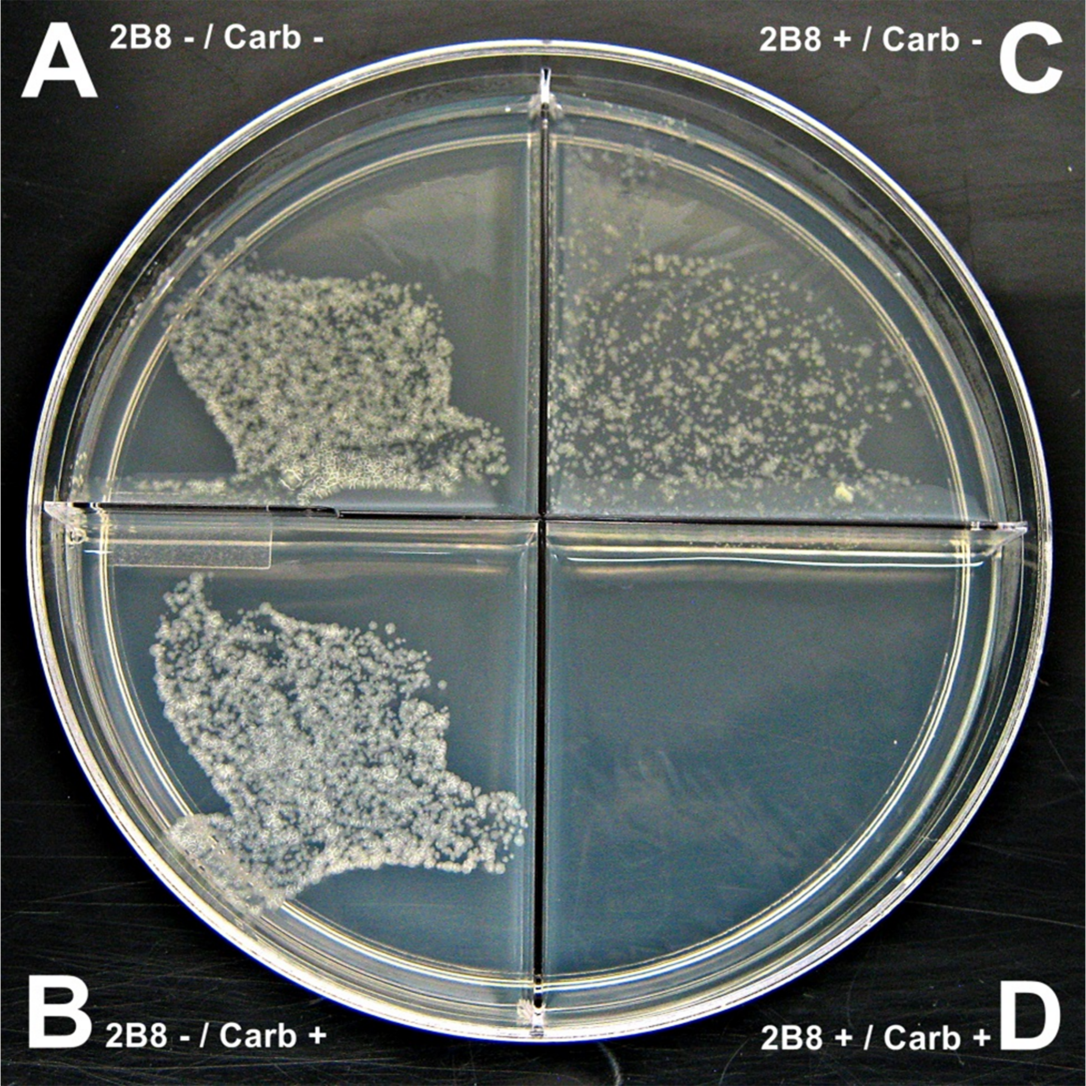
2B8 treated *M*. *tuberculosis* failed to thrive in the presence of carbenicillin. A and B) Untreated *M*. *tuberculosis* H37Rv showing normal growth regardless of the presence of 100 mg/L carbenicillin. C) Growth of *M*. *tuberculosis* H37Rv treated with 125 μM 2B8 was observed in the absence of carbenicillin, albeit to a lesser extent than untreated (A). D) No visible colonies were observed for 2B8 treated *M*. *tuberculosis* H37Rv in carbenicillin-containing agar. The experiment was repeated three separate times in duplicate and a representative photo is shown.

In untreated *M*. *tuberculosis*, the presence or absence of carbenicillin had no effect on growth (Fig 1A and 1B). In contrast, when *M*. *tuberculosis* was treated with 125 μM 2B8, the lead 2-AI compound, the presence of carbenicillin abrogated growth (Fig 1D) whereas growth of 2-AI treated cultures was observed when carbenicillin was excluded from the media (Fig 1C).

### 2-AI treatment reduces MIC of ß-lactams against mycobacteria

Based on the observation that 2B8 treated *M*. *tuberculosis* failed to grow in the presence of carbenicillin, it was hypothesized that 2-AI compounds could potentiate ß-lactams against mycobacteria. Therefore, the MICs of multiple ß-lactams against *M*. *tuberculosis* H37Rv and *M*. *smegmatis* were evaluated in the presence or absence of 2-AI compounds. Compared to the use of ß-lactams alone, combination with 2B8 significantly reduced MIC_95_ values against both *M*. *smegmatis* and *M*. *tuberculosis* (Table 1).

**Table 1:** MIC of ß-lactams against mycobacteria in combination with 2-AI compounds.

*M*. *smegmatis*	MIC	MIC with 5 μM 2B8 (12.5% MIC^1^)	Fold reduction	MIC with 10 μM 2B8 (25% MIC)	Fold reduction	MIC with 20 μM 2B8 (50% MIC)	Fold reduction
Ampicillin	256	256	1	128	2	8	32
Oxacillin	256	256	1	128	2	8	32
Cefotaxime	128	128	1	32	4	1	128
Cefoxitin	16	16	1	8	2	0.5	32
*M*. *tuberculosis*	MIC	MIC with 31.25 μM 2B8 (12.5% MIC^1^)	Fold reduction	MIC with 62.5 μM 2B8 (25% MIC)	Fold reduction	MIC with 125 μM 2B8 (50% MIC)	Fold reduction
Carbenicillin	512	32	16	8	64	4	128
Amoxicillin	512	64	8	16	32	8	64
Ceftazidime	256	128	2	32	8	8	32
Cefotaxime	256	128	2	32	8	4	64
Meropenem	8	2	4	0.25	32	0.0625	128
Penicillin V	512	64	8	16	32	8	64
*M*. *tuberculosis*	MIC	MIC with 31.25 μM RA11 (12.5% MIC^2^)	Fold reduction	MIC with 62.5 μM RA11 (25% MIC)	Fold reduction	MIC with 125 μM RA11 (50% MIC)	Fold reduction
Carbenicillin	512	256	2	128	4	16	32
Amoxicillin	512	512	1	256	2	32	16
Ceftazidime	256	256	1	128	2	64	4
Cefotaxime	256	256	1	128	2	32	8
Meropenem	8	8	1	8	1	1	8
Penicillin V	512	256	2	128	4	32	16

All MIC values are represented as mg/L.

^1^MIC of 2B8 against *M*. *smegmatis* and *M*. *tuberculosis* were 40 μM and 250 μM, respectively.

^2^MIC of RA11 against *M*. *tuberculosis* was 250 μM.

Experiments were carried out at least three independent times and representative data are shown.

For *M*. *smegmatis*, 2B8 reduced the MICs of the four ß-lactams (carbenicillin, amoxicillin, ceftazidime, and meropenem) tested at 25% of the 2B8 MIC (10 μM). MIC reduction was highest when 2B8 was used at 50% of its MIC (20 μM), was 128-fold for cefotaxime and 32-fold for the other tested drugs. For *M*. *tuberculosis*, reduction in ß-lactam MICs became evident at a 2B8 concentration of 12.5% of its MIC (31.25 μM). When used at 50% of its MIC (125 μM), 2B8 reduced the MICs of the five tested ß-lactams at least 32-fold, with the highest fold-reductions of 128-fold observed for carbenicillin and meropenem. RA11, a 2B8 derivative differing only in its alkyl side chains [7], was also tested against *M*. *tuberculosis*. The addition of RA11 also resulted in a reduction in ß-lactam MICs, but to a lesser degree than that for 2B8. For example, the amoxicillin MIC was reduced 64-fold by 2B8 while RA11 only reduced the MIC by 16-fold.

### 2-AI improves bactericidal effect of ß-lactams

Since 2-AI compounds reduced the MICs of ß-lactams against *M*. *tuberculosis*, it was hypothesized that these compounds may augment bactericidal effects. This set of experiments focused on 2B8 because it showed a superior effect over RA11 in the MIC assays. It should be noted that at high concentrations (125 μM), 2B8 treated *M*. *tuberculosis* had impaired growth compared to non-treated culture (S2 Fig). Thus, the bactericidal activity was calculated from non-treated (for ß-lactams only group), 2B8 only treated (2B8/ß-lactams combination group), and clavulanate only treated (clavulanate/ß-lactams combination group) cultures.

**Fig 2:**
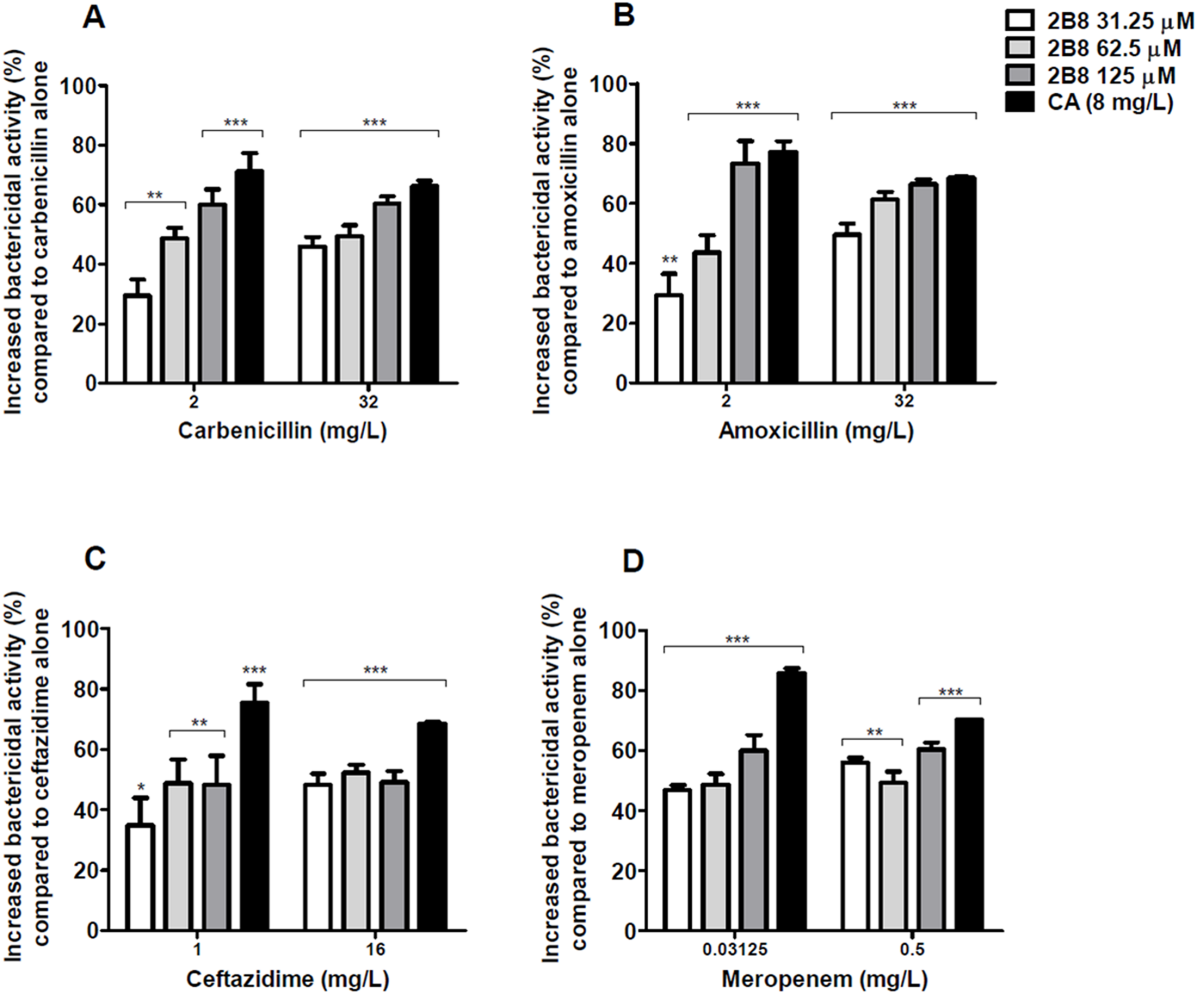
2B8 potentiates mycobactericidal activity of ß-lactams. Bactericidal activity of ß-lactams against *M*. *tuberculosis* H37Rv was significantly increased after 5 days of treatment in combination with 2B8 or clavulanate compared to when ß-lactams were used alone. Statistical significance was determined comparing each group with ß-lactams only group. *p<0.05, **p<0.01, ***p<0.001 by ANOVA. Experiments were carried out three separate times in duplicate and all results were pooled together for statistical analysis.

For the four ß-lactams tested, co-treatment for five days with 2B8 led to a significant increase in bactericidal activity compared to ß-lactams alone. For carbenicillin (2 mg/L), amoxicillin (2 mg/L), and ceftazidime (1 mg/L), a dose-dependent effect was observed with increasing concentrations of 2B8 (Fig 2). The combination of ß-lactams with clavulanate, a widely used ß-lactamase inhibitor, was also evaluated. As expected, improved bactericidal activity was observed when clavulanate was combined with all tested ß-lactams as depicted in Fig 2.

### 2-AI treated *M*. *tuberculosis* cultures have reduced ß-lactamase activity

**Fig 3:**
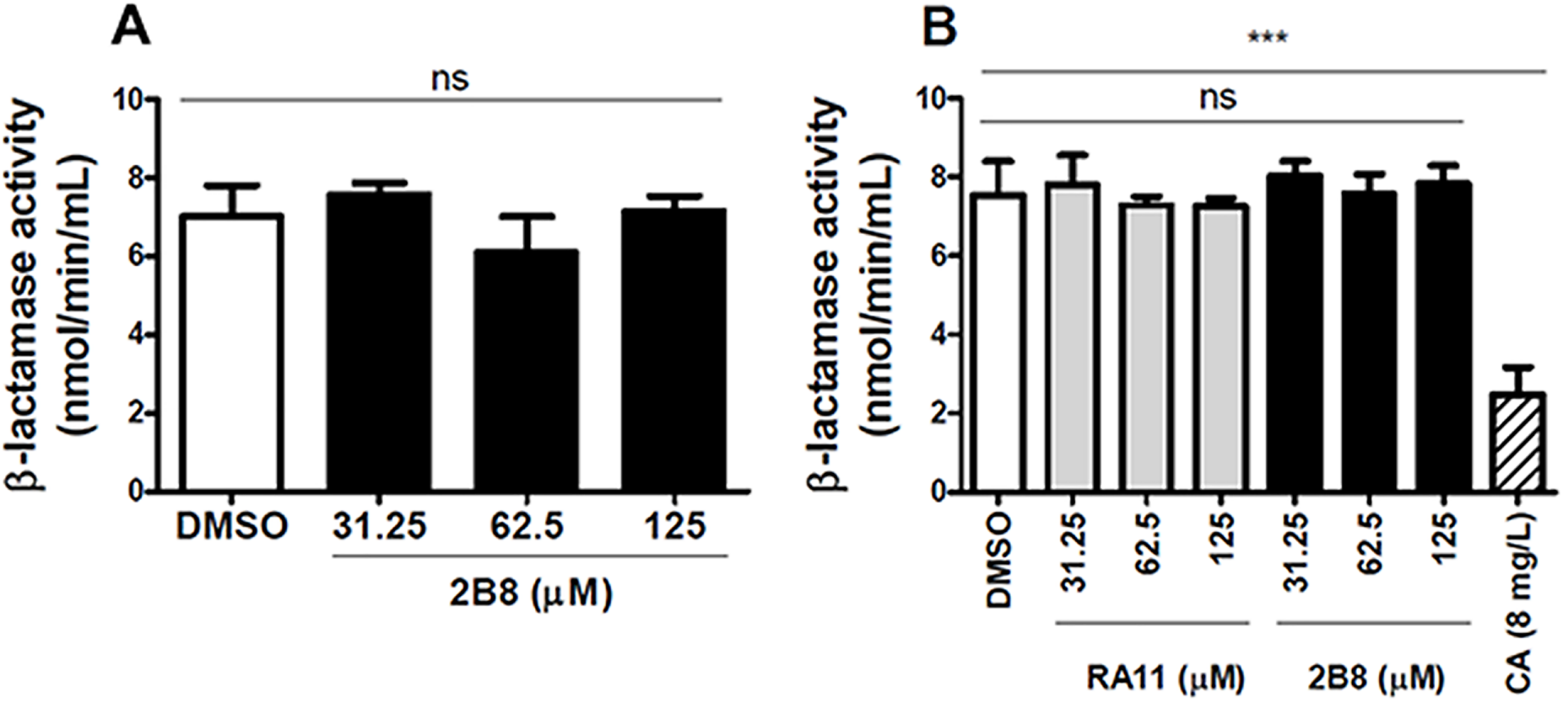
ß-lactamase is not directly inhibited by 2-AI compounds. A) The activity of purified ß-lactamase enzyme from *B*. *cereus* was not affected by the presence of 2B8. B) Clavulanate (CA) significantly decreased ß-lactamase activity in isolated CFP from *M*. *tuberculosis* H37Rv, whereas 2-AI compounds had no effect. Experiments were carried out two separate times in duplicate and representative data are shown. Control: DMSO treated cells. ***p< 0.001 by ANOVA.

An important factor contributing to *M*. *tuberculosis* intrinsic ß-lactam resistance is the synthesis and secretion of ß-lactamase [20, 51, 52]. Indeed, the combination of a meropenem and a ß-lactamase inhibitor such as clavulanate (with or without amoxicillin) has been demonstrated to be effective against *M*. *tuberculosis* both *in vitro* and *in vivo* [22, 33]. Thus, the possibility that 2-AI compounds potentiate ß-lactams by reducing ß-lactamase activity was investigated. To evaluate whether the compounds have a direct effect on the enzyme’s activity, 2-AI compounds were added to either purified *Bacillus* (*B*.) *cereus* ß-lactamase (provided in the colorimetric kit) or *M*. *tuberculosis* CFP, a rich source of mycobacterial specific ß-lactamase [53], and the enzymatic activity was evaluated using the nitrocefin hydrolysis assay. Under these experimental conditions, 2-AI compounds did not directly inhibit ß-lactamase activity (Fig 3A and 3B). As expected, however, clavulanate efficiently inhibited ß-lactamase activity from *M*. *tuberculosis* CFP (Fig 3B).

**Fig 4:**
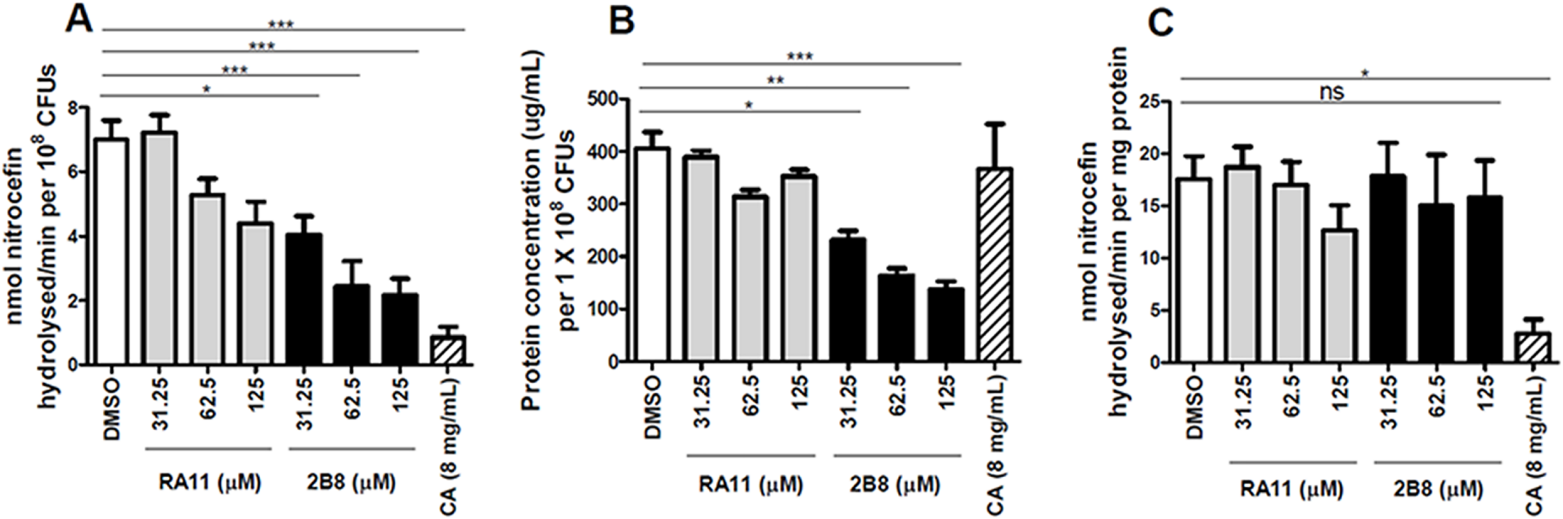
Reduced ß-lactamase activity in 2-AI treated *M*. *tuberculosis* CFP resulting from lower protein secretion. A) After 24 h of treatment, 2B8 (31.25 to 125 μM) treated samples showed significantly decreased ß-lactamase activity in the CFP of *M*. *tuberculosis* H37Rv, whereas DMSO control and RA11 did not. B) Significantly less protein concentration was observed in samples treated with 2B8 (31.25 to 125 μM), but not with clavulanate (CA 8 mg/L). For panels A and B, total nitrocefin hydrolysis activity was normalized to viable bacteria after treatment. C) Except for clavulanate, treatment with 2B8 or RA11 did not reduce ß-lactamase activity in the CFP when normalized to protein concentration. *p<0.05, **p<0.01, ***p<0.001 by ANOVA. Experiments were carried out three separate times in triplicate and representative data are shown.

Alternatively, ß-lactamase activity was measured in CFP obtained from 2-AI treated *M*. *tuberculosis* cultures. *M*. *tuberculosis* cultures treated with RA11 or 2B8 resulted in a dose dependent decrease in ß-lactamase activity after normalization of the data to CFUs (Fig 4A). Consistent with results obtained from the assays described above, 2B8 more effectively reduced ß-lactamase activity than RA11. Reduced ß-lactamase activity in 2-AI treated cultures correlated with a lower total protein concentration present in the CFP of these cultures (Fig 4B). In fact, no differences were observed between control and 2-AI treated cultures when ß-lactamase activity was normalized to protein concentration (Fig 4C). Clavulanate treatment also effectively decreased ß-lactamase activity when data were normalized to viable CFUs (Fig 4A), but did not have any effect in overall protein concentration in the CFP (Fig 4B). Therefore, clavulanate treatment still decreased ß-lactamase activity in the sample even when the data were normalized by total protein concentration (Fig 4C).

### 2-AI treatment alters *M*. *tuberculosis* cell envelope lipid composition

Intrinsic resistance of mycobacteria to ß-lactams has also been attributed to several unique features including the low permeability of the lipid rich cell envelope [54]. It was posited that 2-AI treatment could increase the susceptibility to ß-lactams by altering the mycobacterial cell envelope composition and increasing permeability to this class of antibiotics. To investigate if 2-AI compounds impair mycobacterial cell envelope lipid synthesis or composition, metabolic labeling of mycobacterial lipids was performed with radiolabeled acetate or propionate and relative lipid abundance analyzed by TLC and autoradiogram.

**Fig 5:**
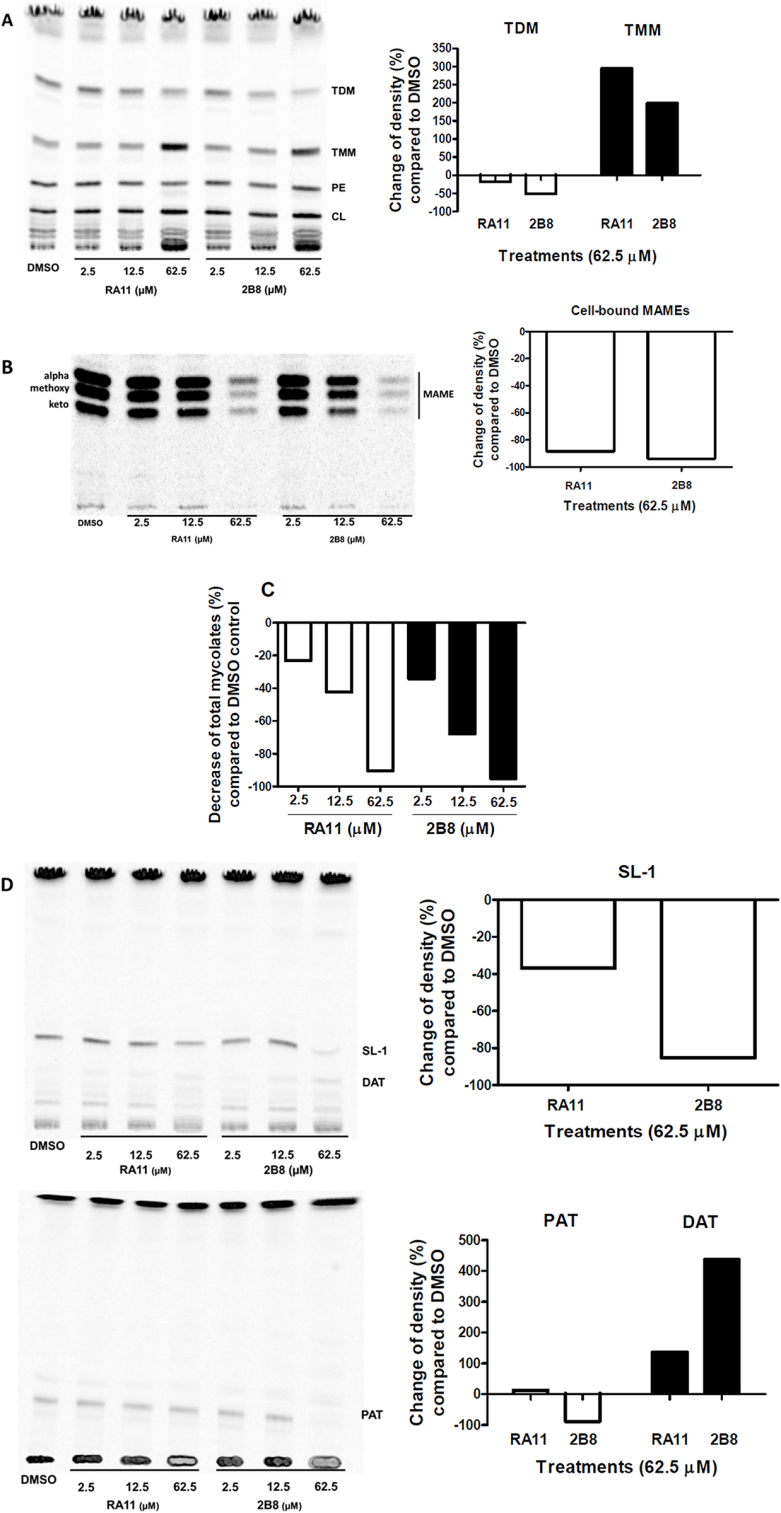
2-AI treatment alters cell envelope lipid composition of *M*. *tuberculosis*. Radioactive metabolic labeling was performed on *M*. *tuberculosis* H37Rv mc^2^ 6206 treated with increasing concentrations of 2-AI compounds (2.5, 12.5, and 62.5 μM) for 24 h. TLCs (left) of a representative experiment are shown with quantified comparison between specific bands as measured by densitometry (right). A) For visualization of TDM, TMM, PE and CL (A), a total of 10,000 cpm was loaded per lane and TLCs were developed in chloroform/methanol/water (20:4:0.5, v/v/v). Treatment with 2-AI compounds resulted in reduced levels of TDM, but increased TMM. B) TLC of cell wall associated mycolates is shown (Left panel). For visualization of MAMEs in cell-bound lipids, hexane/ethyl acetate (95:5, v/v, three developments) solvent system was used and samples were loaded volume to volume. C) Samples treated with 2-AI showed reduced levels of total mycolate biosynthesis (sum of MAMEs from cell-bound, B, and extractable lipids, S3 Fig). D) TLCs were developed in chloroform/methanol/water (90:10:1, v/v/v) for visualization of SL-1 and DAT, and petroleum ether/acetone (92:8, v/v) for visualization of PAT. Treatment with 2-AI compounds reduced the amount of SL-1 and PAT, but increased DAT. All TLCs were quantified by densitometry analysis (right panels). Experiments were carried out three separate times and representative data are shown. PE: phosphatidylethanolamine, CL: cardiolipin.

After 24 h of 2-AI treatment, total radioactive counts from treated samples showed dose dependent decrease, implying reduced biosynthesis of cell envelope extractable lipids (data not shown). Thus, TLC loading was normalized to total radioactive count so that every sample would have equal amounts of labeled total lipids. From TLC analysis of ^14^C acetate labelled extractable lipids (Fig 5A), it was observed that 2-AI treatment led to decreased TDM biosynthesis while accumulating its precursor trehalose monomycolate (TMM). Also, significantly less mycolic acid methyl esters (MAMEs) were extracted from 2-AI treated samples (Fig 5B). Consistent with the MIC assay results, 2B8 treatment resulted in a more dramatic effect than RA11 treatment. Importantly, the biosynthesis of total mycolic acids (determined as the sum of MAMEs from cell-bound and extractable lipids) was reduced in 2-AI treated bacilli (Fig 5C). In the TLC analysis of ^14^C propionate labelled extractable lipids, a significant decrease in sulfolipid-1 (SL-1) and polyacyltrehalose (PAT) biosynthesis in 2B8 treated cultures was observed (Fig 5D). However, diacyltrehalose (DAT), a precursor of PAT, accumulated with 2B8 treatment. Again, 2B8 treatment more pronouncedly affected ^14^C propionate labelled lipids than RA11.

### 2-AI treated *M*. *tuberculosis* becomes hypersensitive to SDS

**Fig 6:**
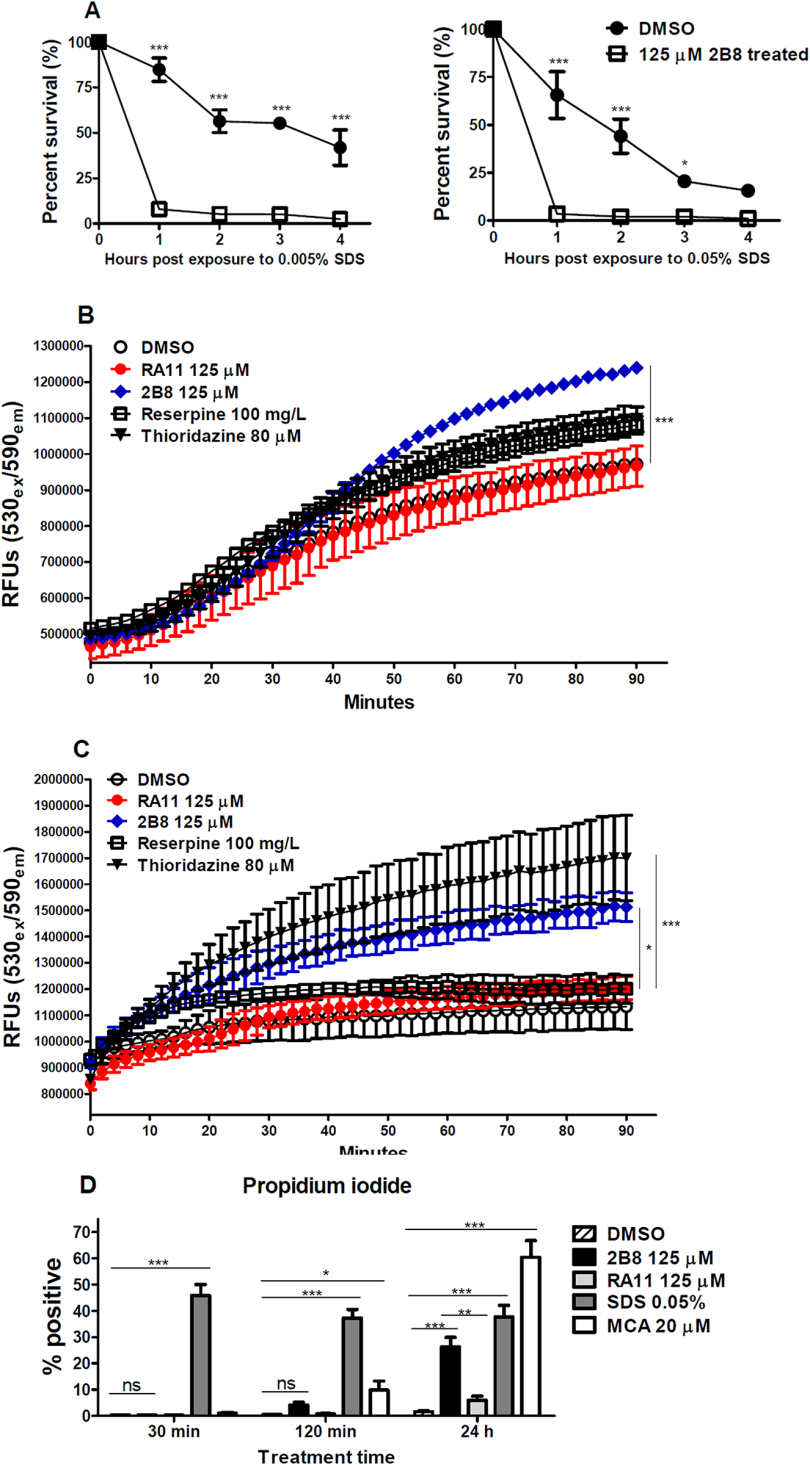
2-AI treatment increased *M*. *tuberculosis* sensitivity to SDS and permeability to nucleic acid staining dyes. A) 2B8 treated *M*. *tuberculosis* H37Rv was significantly more sensitive to killing by SDS at both tested concentration (0.005% and 0.05%). B and C) Compared to DMSO treated culture, 2B8 treated culture showed significantly higher accumulation of EtBr and Sytox Orange after 90 min. 100 mg/L reserpine increased accumulation of EtBr albeit not statistically significant (panel B), while 80 μM thioridazine significantly increased accumulation of Sytox Orange. D) As indicated by increased positive staining with PI analyzed by flow cytometry, SDS acutely (30 min) permeabilized *M*. *tuberculosis* cell membrane, whereas 2B8 and RA11 treatment only compromised the membrane integrity past 2 h. Disruption of cell membrane by 2-AI compounds was more pronounced with 2B8 than RA11 (24 h). MCA treatment also resulted in increased staining with PI after 2 and 24 h of treatment. *p<0.05, **p<0.01, ***p<0.001 by ANOVA, (For EtBr and Sytox Orange accumulation assay, statistical significance marked for 90 min time-point). Experiments were carried out three separate times and representative data are shown.

The lipid rich cell envelope of *M*. *tuberculosis* is known to serve as an impermeable barrier to various chemicals and antibiotics [21]. Based on the alteration in cell envelope lipid composition after 2-AI treatment, it was hypothesized that 2-AI treated *M*. *tuberculosis* would become hypersensitive to membrane-targeting agents such as detergents like SDS. After 24 h treatment with 125 μM 2B8, *M*. *tuberculosis* cultures were exposed to 0.005% or 0.05% SDS. Following SDS exposure, CFUs were enumerated every hour for 4 h. As shown in Fig 6A, significant survival differences were observed between non-treated and 2B8-treated cultures exposed to 0.005% SDS (left panel). The viability of 2B8 treated cultures exposed to SDS dropped more rapidly and to a greater extent than non-treated cultures. As expected, when exposed to SDS at 0.05%, the viability of all the samples declined throughout the course of exposure. However, the decline was more rapid and pronounced in 2B8 treated *M*. *tuberculosis* cultures (Fig 6A, right panel).

### 2-AI treatment increased *M*. *tuberculosis* permeability to multiple nucleic acid staining dyes

Accumulation of nucleic acid staining dyes such as EtBr and Sytox Orange has been used to evaluate *M*. *tuberculosis* cell envelope permeability [38, 39, 55]. Based on the altered cell envelope lipid composition and increased sensitivity to SDS, it was hypothesized that 2-AI treatment increased *M*. *tuberculosis* cell envelope permeability. EtBr or Sytox Orange was applied simultaneously with 2-AI compounds, and the kinetics of fluorescence signal due to dye accumulation was monitored over time.

*M*. *tuberculosis* treatment with 125 μM 2B8 resulted in a time-dependent increase in EtBr fluorescent signal that was significantly higher than untreated control (Fig 6B). This increase was not seen in RA11 treated cultures. Net dye accumulation determined upon completion of the assay was also significantly higher in 2B8 treated cultures (S4A Fig). Reserpine, an inhibitor of the EtBr efflux pump [56], also increased EtBr accumulation within *M*. *tuberculosis*, however it was not statistically significant (Fig 6B). The same trend was observed using Sytox Orange, as both the time-dependent increase and net dye accumulation were significantly elevated in 2B8, but not in RA11 treated cultures (Fig 6C and S4B Fig). In agreement with a recent report [39], thioridazine (an additional positive control) also increased Sytox Orange accumulation (Fig 6C).

Flow cytometry was performed to further evaluate the permeability of 2-AI treated *M*. *tuberculosis* to a third nucleic acid staining dye, PI. Despite sharing a similar chemical structure, PI is a better indicator of plasma membrane integrity than EtBr, due to its additional positive group [57]. In contrast to the above results with EtBr (Fig 6B) and Sytox Orange (Fig 6C), *M*. *tuberculosis* treatment with 2-AI compounds did not acutely increase permeability to PI when evaluated at 30 min and 120 min post-exposure (Fig 6D). However, approximately 30% of *M*. *tuberculosis* became permeable to PI, a classical marker of cell death, upon prolonged exposure to 2B8 for 24 h. Again, *M*. *tuberculosis* membrane disruption was greater for 2B8 than RA11 (Fig 6D). Finally, the two positive controls, SDS and MCA, showed potent *M*. *tuberculosis* cell membrane disrupting capacity, albeit with different kinetics. As expected, a detergent like SDS acutely lysed bacteria within 30 min (Fig 6D), while the effect of MCA only became significant after 24 h.

### 2-AI treatment acutely increases binding of penicillin V and vancomycin

Using fluorescent BODIPY^®^-labeled antibiotics, it was possible to directly measure if 2-AI compounds potentiate ß-lactams by increasing mycobacterial cell envelope permeability and antibiotic accessibility to their respective cell wall targets. To accomplish this, *M*. *tuberculosis* was treated with 2-AI compounds for increasing amount of time (30 min, 120 min and 24 h), stained for 30 min with fluorescent-labeled BODIPY^®^ FL vancomycin or BOCILLIN^®^, and analyzed by flow cytometry. Similar to what was observed with the tested ß-lactams (Table 1), the MIC of vancomycin (S1 Table) and penicillin V against *M*. *tuberculosis* was also lower after exposure to 2-AI compounds.

**Fig 7:**
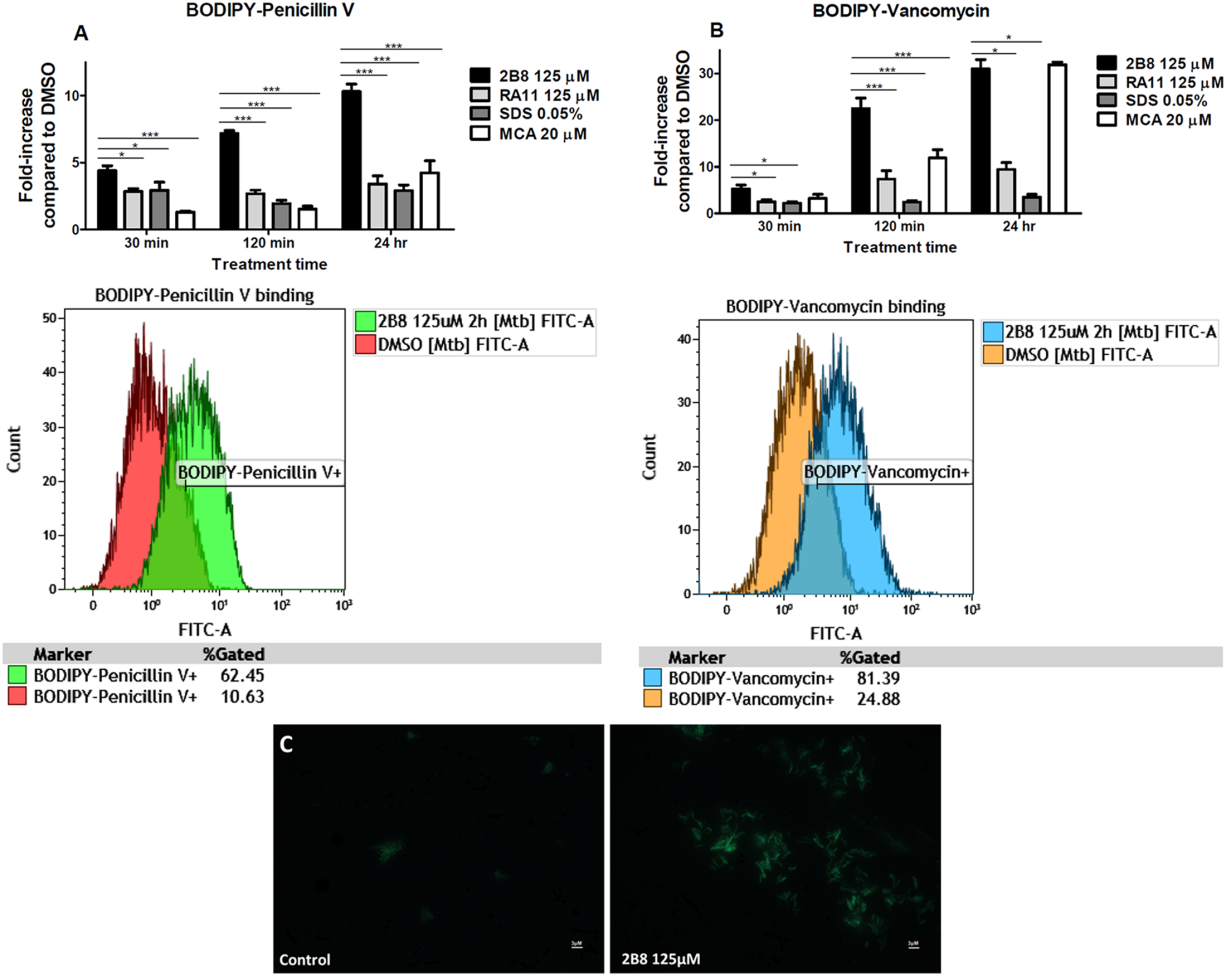
2-AI increased penicillin V and vancomycin binding to *M*. *tuberculosis*. A and B) Representative histogram overlays from flow cytometry analysis (right panels) of BOCILLIN^®^ or BODIPY^®^ FL vancomycin stained *M*. *tuberculosis* after treatment with 125 μM 2B8 or DMSO control. Fold-change staining (left panels) was determined by dividing percent of positively-stained treated bacteria by percent of positively-stained DMSO control bacteria. For BOCILLIN^®^, a time-dependent increase in staining was observed with 2B8, but not with RA11, SDS or MCA treatment. 2B8 treatment also increased staining with BODIPY^®^ FL vancomycin to a significantly higher extent in a time-dependent manner compared to RA11 or SDS treatment. MCA treatment resulted in a time-dependent increase of BODIPY^®^ FL vancomycin staining as expected. C) Fluorescent images (×1000) of *M*. *tuberculosis* stained with BODIPY^®^ FL vancomycin after treatment or not with 125 μM 2B8 for 120 min. Treatment with 2B8 (right panel) resulted in increased number of bacteria with higher fluorescence intensity than DMSO control. *p<0.05, **p<0.01, ***p<0.05 by ANOVA. Experiments were repeated three separate times in triplicates and results were pooled together for statistical analysis. For clarity, significance is only shown for control and 2-AI compound treatment.

There was a significant increase in binding of both fluorescent penicillin V (Fig 7A and S5 Fig) and vancomycin (Fig 7B and S5 Fig) to *M*. *tuberculosis* compared to controls after 30 min treatment with 2B8. For both fluorescent antibiotics, binding to treated *M*. *tuberculosis* increased proportionally to treatment duration. Treatment with RA11 also increased antibiotic binding to *M*. *tuberculosis* but the extent was significantly less compared to 2B8. The binding of fluorescent vancomycin to *M*. *tuberculosis* increased when treated with MCA (Fig 7B), as expected considering meropenem’s inhibition of the mycobacterial D,D-carboxypeptidase that cleaves vancomycin’s target, the D-Ala-D-Ala peptide motif [40]. However, this effect was delayed and only became evident after 24 h treatment. Moreover, despite being a potent disruptor of the cell membrane (Fig 7A and 7B), SDS only minimally increased binding of either BODIPY^®^ FL vancomycin or BOCILLIN^®^ to *M*. *tuberculosis*, suggesting that direct membrane disruption does not underpin activity. The specificity of BODIPY^®^ FL vancomycin’s staining was confirmed by a competitive inhibition assay and fluorescent microscopy. Increasing amounts of unlabeled vancomycin competitively inhibited binding of BODIPY^®^ FL vancomycin to 2B8 treated *M*. *tuberculosis* (S6 Fig). These data confirm that the fluorescent drug is binding specifically to its cognate target, rather than non-specifically through the BODIPY^®^ moiety. Furthermore, in accordance with previous publications [58, 59], punctate staining predominantly at the mycobacterial poles was observed when *M*. *tuberculosis* was stained with BODIPY^®^ FL vancomycin (Fig 7C). Consistent with the flow cytometry results, 2B8 treatment increased the number of stained bacteria and fluorescence intensity versus DMSO-treated control (Fig 7C). BOCILLIN^®^’s fluorescence was not bright enough for microscopy (data not shown).

### *M*. *tuberculosis* transcriptional responses to 2B8

**Fig 8:**
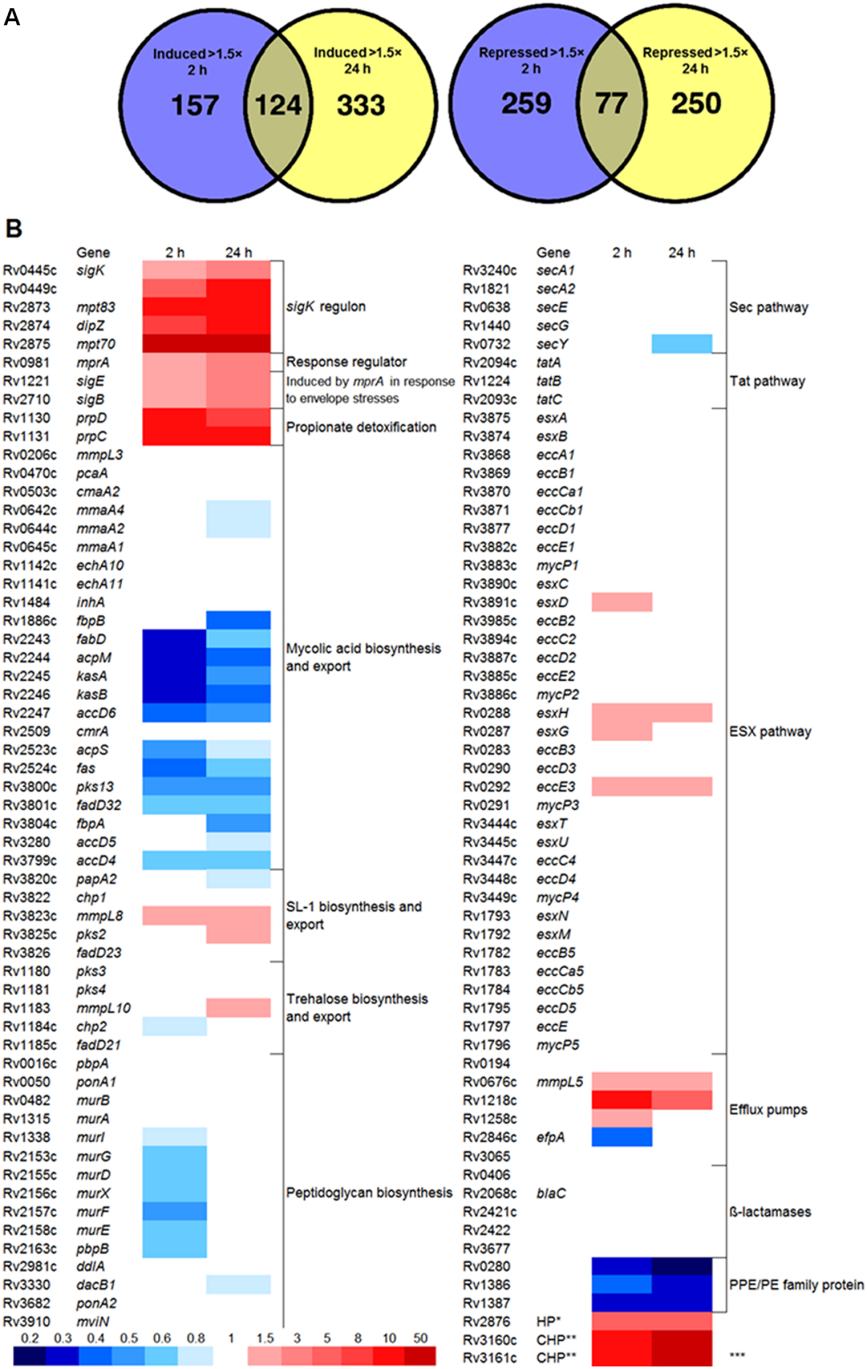
Transcriptional responses of *M*. *tuberculosis* to 2B8. RNA-seq analysis was performed on *M*. *tuberculosis* H37Rv treated with DMSO (untreated control) or with 125 μM 2B8 for 2 and 24 h. Induced and repressed genes were defined as >1.5-fold change in expression compared to the untreated control, q<0.05. A) Venn diagram showing number of genes induced or repressed after 2 and 24 h. B) Heat map showing differential transcriptional regulation of selected relevant genes. Genes that did not make the 1.5-fold cut-off and statistical significance (q<0.05) were considered no change (1 in fold-change scale, white color). *Hypothetical protein, **Conserved hypothetical protein, ***Upregulated in response to triclosan.

To further characterize the impact of 2-AI compounds on *M*. *tuberculosis* physiology, transcriptional profiling of *M*. *tuberculosis* exposed to 2B8 for 2 and 24 hours was performed. *M*. *tuberculosis* H37Rv was treated with 125 μM 2B8 and following 2 or 24 h of treatment, RNA was isolated and transcriptional profiles were determined by RNA-sequencing (RNA-seq). Upregulated or downregulated genes (>1.5-fold with a q <0.05) were identified after 2 h (S3 Table) or 24 h (S4 Table) post-treatment. To identify genes with both early and sustained differential gene expression, the gene lists at 2 and 24 h (S5 Table) were compared for common differentially regulated genes (Fig 8A). At both time points, 124 genes were induced and 77 genes were repressed (>1.5-fold, q<0.05). Genes encoding for several transcriptional regulators including the alternative sigma factors *sigB*, *sigE* and *sigK* and the response regulator *mprA* were induced (1.5 to 1.8-fold for 2 h, 2 to 3.7-fold for 24 h) (Fig 8B). 2B8 treatment enhanced expression of SigK regulated genes including *mpt83*, *dipZ*, *mpt70*, and *rv0449c* [60], supporting that the SigK regulon was induced in a sustained manner by 2B8 (Fig 8B). Other genes of interest that were strongly induced at both time-points include: *prpCD* which is proposed to play a role in propionate detoxification [61], *rv3160c* and *rv3161c*, a putative dioxygenase and its regulator, previously shown to be strongly regulated by triclosan and suggested to be involved in degradation of arenes [62]. Finally, it was noted that *mmpL8* (2 and 24 h) and *mmpL10* (24 h) involved in SL-1 and acyl-trehalose export, respectively [63, 64], were induced by 2B8 treatment (Fig 8B).

The downregulated genes at both time-points show a strong signature for inhibition of genes associated with mycolic acid biosynthesis (Fig 8B) including *fas*, *fabD*, *acpM*, *kasAB*, *pks13* and *fadD32*. This result is consistent with 2-AI-mediated modulation of mycolic acids biosynthesis determined by metabolic labeling experiment (Fig 5A and 5B). Multiple genes involved in peptidoglycan biosynthesis such as the *mur* family were repressed in response to treatment. However, genes involved in protein secretion were not generally modulated by 2B8. The *blaC* and other recently identified genes [65, 66] encoding for ß-lactamases were also unaffected (Fig 8B). Finally, one of the most significantly downregulated genes was *rv0280*, which encodes for a member of the PE/PPE family with no known function to date, but previously reported to be downregulated in a *phoP* mutant strain [67].

## Discussion

While investigating the mechanism of action of 2-AI compounds against non-replicating mycobacteria *in vitro*, these compounds were serendipitously observed to potentiate ß-lactam antibiotics against *M*. *tuberculosis*. Specifically, 2-AI treated *M*. *tuberculosis* failed to grow on agar containing carbenicillin. 2-AI compounds were subsequently shown to effectively reduce MICs and bactericidal concentrations of multiple ß-lactams against *M*. *tuberculosis*. It is noteworthy that the effect of 2-AI was not limited to a specific class of ß-lactams. Indeed, 2-AI’s effect was observed (albeit to a different degree) across all ß-lactams tested including carboxypenicillin (carbenicillin), aminopenicillin (amoxicillin), third generation cephalosporin (ceftazidime), and carbapanem (meropenem). In essence, 2-AI compounds effectively nullified *M*. *tuberculosis* intrinsic ß-lactam resistance.

Based upon this observation, the goals of this study were to determine if 2-AI compounds circumvents *M*. *tuberculosis* intrinsic resistance to ß-lactams and if so, to elucidate the mechanism of action. It is reported herein that selected 2-AI compounds were effective in reducing ß-lactam MICs against *M*. *tuberculosis* while also improving their bactericidal activity. Mechanistic studies have revealed that 2-AI compounds achieve this response by at least two distinct mechanisms: a) increasing *M*. *tuberculosis* cell envelope permeability and accessibility of cell wall targeting drugs and b) reducing *M*. *tuberculosis* ß-lactamase secretion.

Several lines of evidence indicate that a major mechanism contributing to ß-lactam potentiation by 2-AI compounds is the alteration of the mycobacterial cell envelope and an increase in the accessibility of ß-lactams to their target [12, 13, 16, 21, 54, 55]. Indeed, as early as 30 min post-treatment with 2-AI compounds, increased *M*. *tuberculosis* permeability to a fluorescent ß-lactam or glycopeptide antibiotic, such as penicillin V and vancomycin, respectively was observed. In order to reach their targets located within the periplasmic space delimited between the outer and inner cell membrane, these antibiotics first have to diffuse through the outer cell membrane [19, 24, 68, 69]. It is well documented in both mycobacteria and Gram-negative bacteria that the presence of an outer cell membrane acts as a permeability barrier to limit the diffusion of hydrophilic molecules [55, 70–73]. Specifically, in mycobacteria, the outer cell membrane impermeability is further enforced by the presence of mycolic acids and complex lipids such as TDM and PDIM in the inner and outer leaflets [24]. The fact that 2B8-treated *M*. *tuberculosis* was readily stained with a bulky antibiotic (such as BODIPY^®^ FL vancomycin) within 30 min, in contrast to multiple hours usually required to stain mycobacteria [58, 74], strongly suggests that 2B8 increases the mycobacterial outer cell membrane permeability. This hypothesis was further supported by the acute (90 min) increased permeability of 2B8-treated *M*. *tuberculosis* to nucleic acid staining dyes EtBr and Sytox Orange, which are routinely used as indicators of mycobacterial cell envelope integrity [54, 55]. The ability to increase the outer membrane permeability could potentially be attributed to 2-AI compound’s alkyl chain(s) and could explain the different structure-function relationship of several related 2-AI compounds sharing the same 2-aminoimidazole polar head group but derivatized with distinct apolar, alkyl chains. As suggested by the results with fluorescent antibiotics and nucleic acid staining dyes, 2B8’s branched, albeit short alkyl chain induces a superior effect on the mycobacterial outer cell membrane permeability compared to RA11’s straight alkyl chain containing 11 carbons. Derivatization of the 2-aminoimidazole group with a straight 13-carbon alkyl chain (such as in another 2B8 derivative, RA13), completely abrogated the compound’s activity in the mycobacterial biofilm dispersion assay [7], as well as in some of the assays performed herein (data not shown). This dependence on a critical chain length and structure of the hydrophobic tail could explain why SDS, with a straight 12-carbon acyl chain, failed to dramatically potentiate ß-lactams (S2 Table) or increase binding of fluorescent antibiotics to *M*. *tuberculosis* despite being a potent inner cell membrane disruptor (as determined by PI staining). Meanwhile, mycobacterial inner cell membrane disruption with 2B8 was only observed for a small fraction of bacilli after 24 h. In contrast, Stowe et al. previously reported acute and dramatic *A*. *baumannii* lysis in the presence of reverse-amide 2-AIs [12]. Beyond the role played by their different hydrophobic tails, opposing outcomes induced by 2-AI compounds or SDS could also be determined or modulated by their respective polar head group and charge, which remains to be investigated. Collectively, these results indicate ß-lactam potentiation by 2-AI compounds is uncoupled from direct inner cell membrane disruption.

Further evidence that 2-AI compounds affect the mycobacterial cell envelope was obtained from metabolic labeling experiments evaluating *M*. *tuberculosis* cell envelope lipid composition, as well as from transcriptional responses to 2B8. 2-AI treated *M*. *tuberculosis* had a dramatic decrease in mycolic acids covalently esterified to the cell wall (MAMEs), likely resulting from a combination of: a) reduced biosynthesis, b) defect in TMM export, and c) decreased crosslinking to arabinose residues in the mycolyl-arabinogalactan-peptidoglycan (mAGP) complex. Genes encoding for FAS-I (synthesizes mycolic acid’s α-alkyl chain), FAS-II (synthesizes mycolic acid’s meromycolate chain) and pks13 (condenses both chains to form mycolic acids) [75, 76], were among the most significantly down-regulated genes at 2 h post-treatment. Furthermore, mycolic acid export was also compromised by 2B8 treatment as suggested by the inverse relationship between high TMM (precursor) and low TDM (end product) levels. However, this defect was not a consequence of reduced *mmpL3* transcription, recently identified to encode for a TMM transporter [77]. Finally, by down-regulating expression of two members of the mycolyl-transferase complex (*fbpA* and *fbpB*), which catalyze mycolic acid-arabinose linkage [78], 2B8 could further compound the deficit of mycolic acid-dependent fortification of the mycobacterial cell wall core. Trehalose-based lipids such as SL-1 and PAT were also less abundant in 2-AI treated bacilli, while PAT’s precursor, DAT, accumulated. Again, this deficit was not due to decreased transcription of their cognate transporters. In fact, transcription of *mmpL8* and *mmpL10*, encoding for SL-1 [63] and DAT transporter [64], respectively, was up-regulated. The fact that 2-AI treatment leads to mycobacterial accumulation of unexported lipid precursors without decreased transporter transcription, suggest that the defect is elsewhere, perhaps at the level of proton motive force (PMF) generation required for MmpL-catalyzed transport of related lipids [79]. This line of research is currently being evaluated. Finally, it was recently shown that methyl-branched lipid biosynthesis acts as a propionate sink to limit its toxicity [61, 80, 81]. Interestingly, both *prpC* and *prpD* of the methyl-citrate cycle that plays a role in detoxifying propionate were induced 17- and 34-fold, respectively, following 2 h of 2B8 treatment. This strong induction suggests that 2B8 may result in propionate toxicity, possibly as part of changes to the cell envelope. Altogether, beyond the acute increased outer cell membrane permeability discussed above, alterations in the biosynthesis and/or export of *M*. *tuberculosis* cell envelope lipids likely contributes to ß-lactam potentiation and enhanced SDS sensitivity induced by 2-AI compounds.

Bacteria respond to cell envelope stress by modulating their transcriptome through the activation of transcription regulators. Consistent with this, at 2 h after treatment, 2B8 induced several transcriptional regulators including the alternative sigma factors *sigB*, *sigE*, and *sigK* and the response regulator *mprA*. Notably, the two component regulatory system MprAB regulates *sigB* and *sigE* [82] in response to envelope stresses, such as SDS or Triton X-100 treatment, which is consistent with 2-AI promoting envelope stress and stimulating the MprAB regulatory network. Both SigE and SigK belong to the family of extracytoplasmic-function sigma factors (ECF), kept inactive/transcriptionally silent by remaining tethered to a transmembrane protein, an anti-sigma factor [83–85]. In the presence of extracellular stress, proteolytic cleavage of the anti-sigma factor releases the cognate sigma factor to become transcriptionally active and regulate the expression of multiple genes. Particularly interesting was the upregulation of *sigK* and SigK regulated genes such as *mpt70*, *mpt83*, *dipZ*, and *rv0449c* [60], supporting that the SigK regulon is induced in a sustained manner by 2B8. Unfortunately, not much is known about the biological role of the SigK regulon, besides the fact the *mpt70/83* are highly immunogenic proteins and their expression levels significantly differ between members of the *M*. *tuberculosis* complex [60, 86]. Two redox-sensitive cysteine residues were recently suggested to regulate the transcriptional activity of SigK by altering the interaction with RskA, the cognate anti-sigma factor [87]. This is a recurring mechanism regulating other ECF such as SigF and SigL [88, 89], however the upregulation of these sigma factors or their regulons in response to 2B8 was not observed. Thus, the mechanism leading to the specific upregulation of the SigK regulon by 2B8 is still unclear.

The *M*. *tuberculosis blaC* gene encodes for a highly active class A ß-lactamase, which significantly contributes to *M*. *tuberculosis* intrinsic ß-lactam resistance [20, 51]. Therefore, studies have attempted to circumvent this resistance by combining ß-lactams with a ß-lactamase inhibitor such as clavulanate. This combination has indeed proven to be promising against *M*. *tuberculosis* [22, 32, 90]. Thus, it was important to determine if 2-AI compounds potentiate ß-lactams by interfering with any aspect of *M*. *tuberculosis* ß-lactamase function. 2-AI compounds did not directly inhibit ß-lactamase activity like the classical inhibitor clavulanate, supporting our recently published results obtained with additional compounds derived from the 2-AI scaffold [91]. This result was expected considering 2-AI compounds similarly potentiated ß-lactams regardless of their respective susceptibility to ß-lactamase degradation, suggesting ß-lactamase inhibition was not the mechanism driving potentiation. Comparable MIC fold-reduction was observed for carbenicillin/amoxicillin/penicillin V (early generation of ß-lactams highly susceptible to ß-lactamase) and meropenem (a carbapenem with superior resistance to ß-lactamase). Furthermore, ß-lactam potentiation by 2B8 was not due to down-regulation of genes encoding for ß-lactamases such as *blaC* (*rv2068c*), *rv0406*, *rv2421c*, *rv2422*, *and rv3677*. or those encoding for the twin-arginine protein translocating system (tat), responsible for BlaC secretion [92]. However, reduced ß-lactamase activity in the CFP of 2B8 treated *M*. *tuberculosis*, correlated with reduced protein concentration in this fraction. As 2B8 did not alter the transcription of genes encoding for other major *M*. *tuberculosis* protein secretion systems (namely SecA, SecA2 or type VII), it is currently being explored if these compounds affect other parameters such as mycobacterial bioenergetics required for protein secretion and lipid export, as described above. Thus, it is concluded that reduced ß-lactamase secretion could contribute in part to ß-lactam potentiation by 2-AI compounds.

Other aspects of *M*. *tuberculosis* intrinsic resistance to ß-lactams that could be affected by 2-AI treatment but were not directly evaluated, include the role of efflux pumps, peptidoglycan structure and PBPs or L,D-transpeptidases involved in peptidoglycan crosslinking [16, 93]. However, through transcriptional profiling it was possible to rule out decreased gene expression of efflux pumps, PBPs or L,D-transpeptidases as a mechanism explaining ß-lactam potentiation by 2-AI compounds. In fact, 2B8 induced *rv1218c* and *rv1258c*, previously shown to be specifically involved in ß-lactam resistance [56, 94], as well as other efflux pumps associated with resistance to bedaquiline and clofazimine (*mmpL5*) [95]. Nevertheless, it cannot be excluded that 2-AI compounds have an indirect effect on ß-lactam efflux pumps, via alteration of mycobacterial bioenergetics as discussed above. Finally, as suggested by lower expression levels for *mur* genes, 2B8 could potentially be reducing the amount of uncrosslinked peptidoglycan precursors, the substrates for PBPs and L,D-transpeptidases. Ultimately, in the face of dwindling amounts of peptidoglycan precursors, PBPs and L,D-transpeptidase inhibition by ß-lactams would have additive catastrophic effects.

Taken together, the data suggests that 2-AI compounds potentiate ß-lactams by affecting *M*. *tuberculosis* cell envelope integrity and ß-lactamase secretion. Evidently, a limitation in this study was the high concentration of 2-AI compounds required for most of the assays, including ß-lactam potentiation which started to occur at 31.25 μM. Through medicinal chemistry, a second generation of 2-AI compounds is being developed with similar activity against *M*. *tuberculosis*, at lower concentrations. Importantly, findings in this study are a proof of concept of the possibility of potentiating ß-lactams with 2-AI compounds as a novel therapeutic regimen for drug susceptible and multi-drug resistant TB.

## Supporting information

S1 Fig Structures of 2B8 and RA11. (TIF)

S2 Fig 2B8 affects normal growth of *M*. *tuberculosis* H37Rv. *M*. *tuberculosis* H37Rv was plated on 7H11 agar after five days of culture with or without clavulanate or increasing concentrations of 2B8, and CFUs were enumerated three weeks after. Compared to control, CFUs from cultures containing 2B8 were significantly lower, suggesting that 2B8 affects normal growth of *M*. *tuberculosis* by itself. In contrast, clavulanate did not affect bacterial growth. *p<0.05 by ANOVA.
(TIF)

S3 Fig 2-AI treatment reduces MAMEs from extractable lipids in *M*. *tuberculosis*. MAMEs were derived from extractable lipids of [1-^14^C] acetate-labeled *M*. *tuberculosis* H37Rv mc^2^ 6206 strain and analyzed by TLC as described in Fig 5B. Treatment with 2-AI compounds resulted in reduced MAMEs from extractable lipids. Experiments were carried out three separate times and representative data are shown.
(TIF)

S4 Fig 2B8 treatment increased *M*. *tuberculosis* net accumulation of nucleic acid staining dyes. 2B8 treated *M*. *tuberculosis* accumulated both EtBr (A) and Sytox Orange (B) significantly more than untreated. Reserpine and thioridazine increased net accumulation of EtBr, but it was not statistically significant (A). Thioridazine significantly increased accumulation of Sytox Orange (B). *p<0.05, **p< 0.01 by ANOVA.
(TIF)

S5 Fig 2B8 acutely increases binding of BODIPY^®^ FL vancomycin and BOCILLIN^®^ to *M*. *tuberculosis* while having no effect on cell membrane integrity. A) Representative scatter plot of gated population for *M*. *tuberculosis*. B and C) Representative histogram overlays of unstained, DMSO and 125 μM 2B8 treated *M*. *tuberculosis* after staining with BODIPY^®^ FL vancomycin (B) or BOCILLIN^®^ (C). Treatment with 125 μM 2B8 for 2 h resulted in significantly increased binding of both fluorescent vancomycin and penicillin V to *M*. *tuberculosis*. D) In contrast, this treatment did not significantly increase *M*. *tuberculosis* permeability to PI.
(TIF)

S6 Fig Unlabeled vancomycin competitively inhibits binding of BODIPY^®^ FL vancomycin to *M*. *tuberculosis*. Prior to staining with BODIPY^®^ FL vancomycin, unlabeled vancomycin at three different concentrations (50×, 100×, and 500× the amount of BODIPY^®^ FL vancomycin) was added to *M*. *tuberculosis* treated for 2 h with 2B8. Representative histogram overlay of 2B8 treated *M*. *tuberculosis* with or without addition of 500× unlabeled vancomycin is shown (left panel). When 100× or 500× unlabeled vancomycin was added to 2B8 treated samples, binding of BODIPY^®^ FL vancomycin was significantly inhibited (right panel). ***p< 0.001 by ANOVA. Experiments were done three separate times and all individual samples were pooled together for statistical analysis.
(TIF)

S1 Table MIC of vancomycin against 2B8 treated *M*. *tuberculosis* H37Rv. (DOCX)

S2 Table MIC of ß-lactams against SDS treated *M*. *tuberculosis* H37Rv. (DOCX)

S3 Table Differentially regulated *M*. *tuberculosis* genes after 2 h treatment with 2B8 125 μM. (XLSX)

S4 Table Differentially regulated *M*. *tuberculosis* genes after 24 h treatment with 2B8 125 μM. (XLSX)

S5 Table Complete list of *M*. *tuberculosis* genes after 2 and 24 h treatment with 2B8 125 μM. (XLSX)
