# Supplementary figures and images for: 2-aminoimidazoles potentiate ß-lactam antimicrobial activity against Mycobacterium tuberculosis by reducing ß-lactamase secretion and increasing cell envelope permeability
Source: PLoS One. 2017 Jul 27;12(7):e0180925. doi: 10.1371/journal.pone.0180925 (PMC5547695; doi:10.1371/journal.pone.0180925)

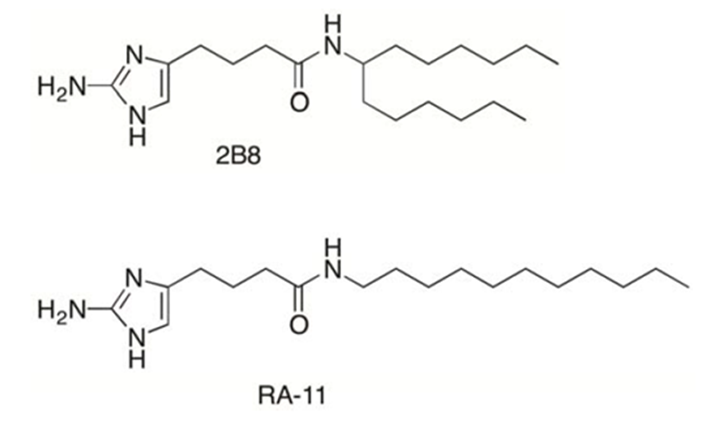

Supplement: S1 Fig — (TIF) [file pone.0180925.s001.tif]

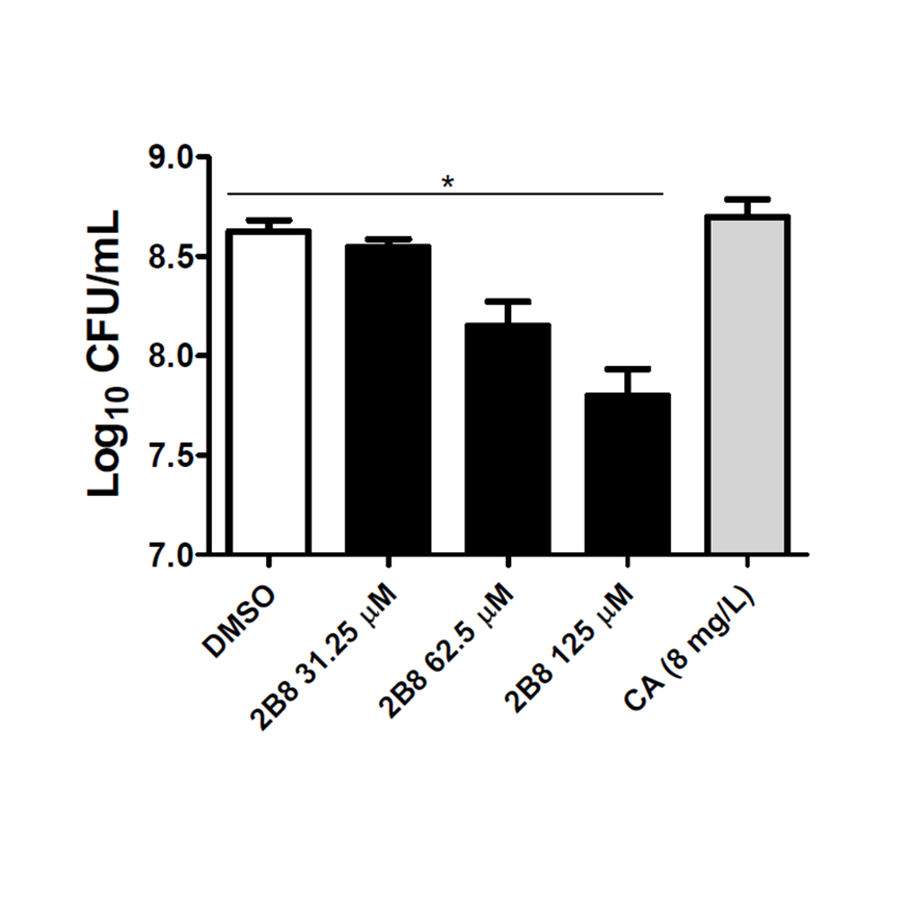

Supplement: S2 Fig — M. tuberculosis H37Rv was plated on 7H11 agar after five days of culture with or without clavulanate or increasing concentrations of 2B8, and CFUs were enumerated three weeks after. Compared to control, CFUs from cultures containing 2B8 were significantly lower, suggesting that 2B8 affects normal growth of M. tuberculosis by itself. In contrast, clavulanate did not affect bacterial growth. *p<0.05 by ANOVA. (TIF) [file pone.0180925.s002.tif]

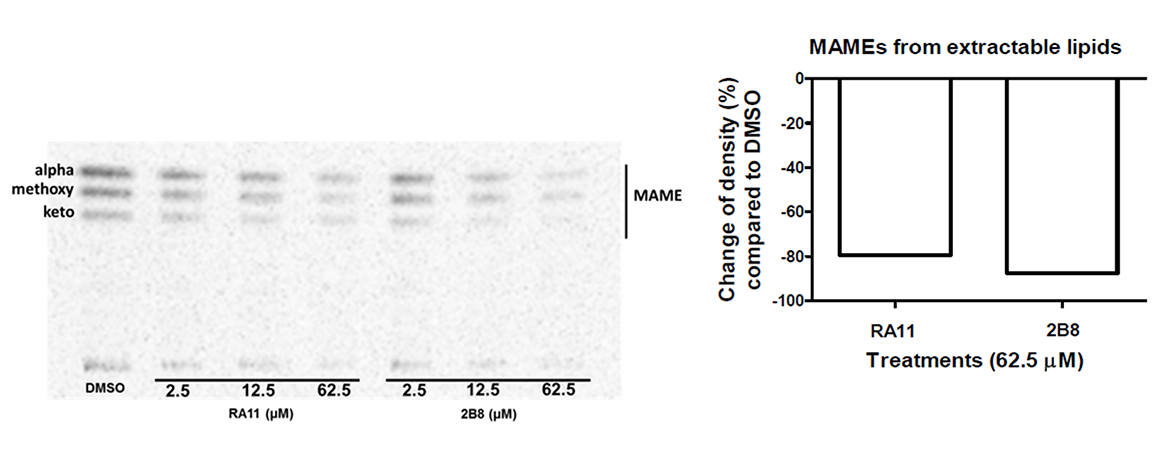

Supplement: S3 Fig — MAMEs were derived from extractable lipids of [1-14C] acetate-labeled M. tuberculosis H37Rv mc2 6206 strain and analyzed by TLC as described in Fig 5B. Treatment with 2-AI compounds resulted in reduced MAMEs from extractable lipids. Experiments were carried out three separate times and representative data are shown. (TIF) [file pone.0180925.s003.tif]

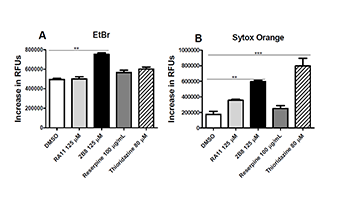

Supplement: S4 Fig — 2B8 treated M. tuberculosis accumulated both EtBr (A) and Sytox Orange (B) significantly more than untreated. Reserpine and thioridazine increased net accumulation of EtBr, but it was not statistically significant (A). Thioridazine significantly increased accumulation of Sytox Orange (B). *p<0.05, **p< 0.01 by ANOVA. (TIF) [file pone.0180925.s004.tif]

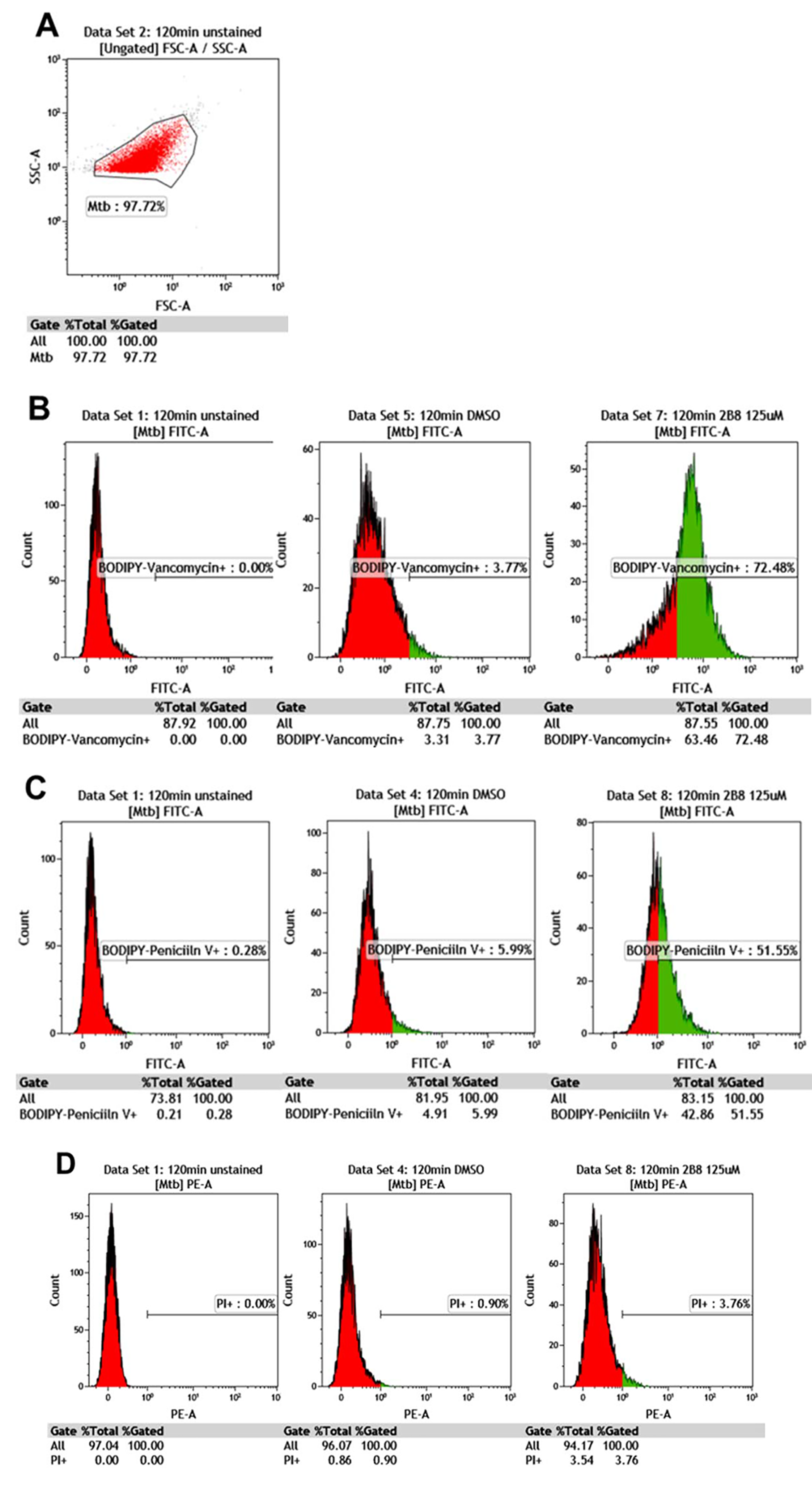

Supplement: S5 Fig — A) Representative scatter plot of gated population for M. tuberculosis. B and C) Representative histogram overlays of unstained, DMSO and 125 μM 2B8 treated M. tuberculosis after staining with BODIPY® FL vancomycin (B) or BOCILLIN® (C). Treatment with 125 μM 2B8 for 2 h resulted in significantly increased binding of both fluorescent vancomycin and penicillin V to M. tuberculosis. D) In contrast, this treatment did not significantly increase M. tuberculosis permeability to PI. (TIF) [file pone.0180925.s005.tif]

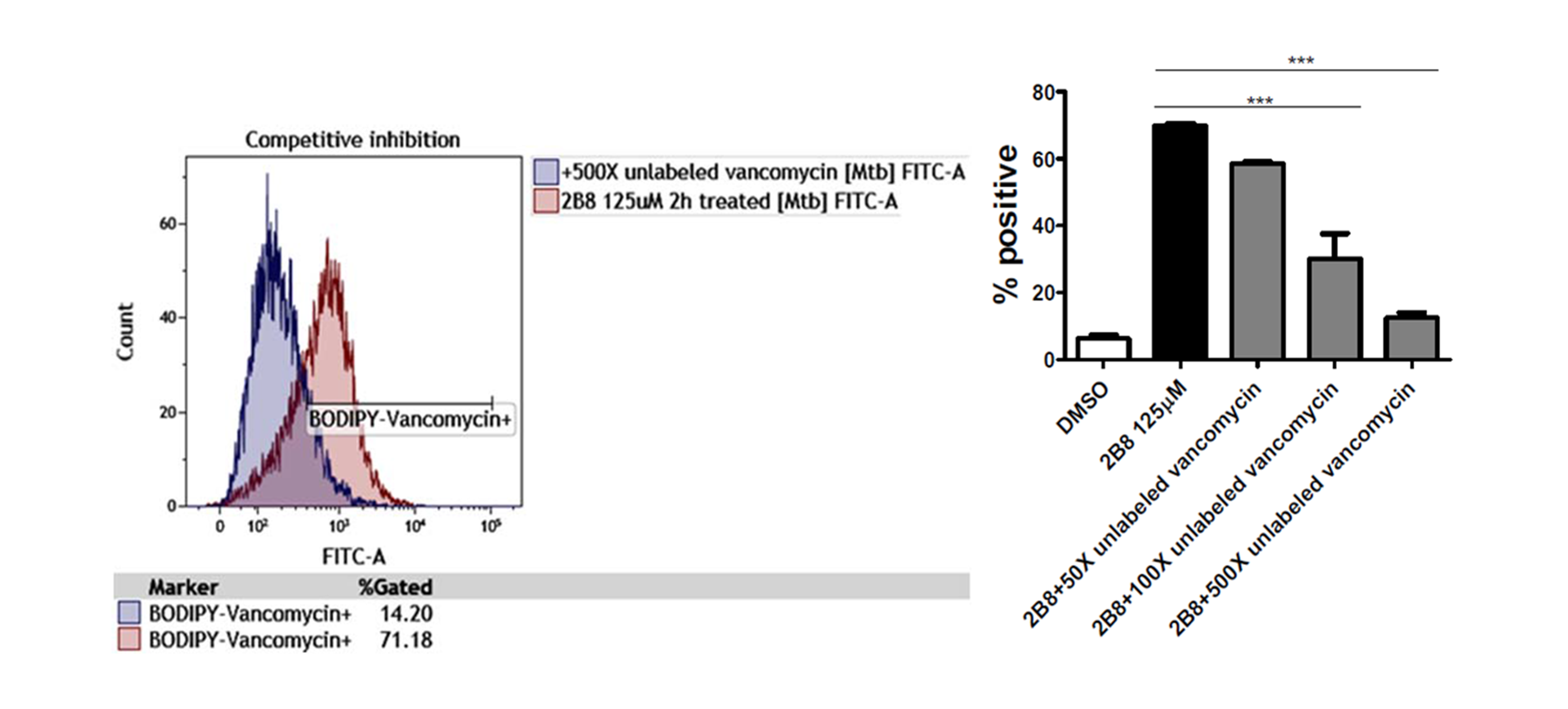

Supplement: S6 Fig — Prior to staining with BODIPY® FL vancomycin, unlabeled vancomycin at three different concentrations (50×, 100×, and 500× the amount of BODIPY® FL vancomycin) was added to M. tuberculosis treated for 2 h with 2B8. Representative histogram overlay of 2B8 treated M. tuberculosis with or without addition of 500× unlabeled vancomycin is shown (left panel). When 100× or 500× unlabeled vancomycin was added to 2B8 treated samples, binding of BODIPY® FL vancomycin was significantly inhibited (right panel). ***p< 0.001 by ANOVA. Experiments were done three separate times and all individual samples were pooled together for statistical analysis. (TIF) [file pone.0180925.s006.tif]
