# Supplementary material for: 2-aminoimidazoles potentiate ß-lactam antimicrobial activity against Mycobacterium tuberculosis by reducing ß-lactamase secretion and increasing cell envelope permeability
Source: PLoS One. 2017 Jul 27;12(7):e0180925. doi: 10.1371/journal.pone.0180925 (PMC5547695; doi:10.1371/journal.pone.0180925)
Supplement: S1 Table — (DOCX) [file pone.0180925.s007.docx]

|  |  | MIC with 31.25 µM 2B8 | Fold | MIC with 62.5 µM 2B8 | Fold | MIC with 125 µM 2B8 | Fold |  |
| --- | --- | --- | --- | --- | --- | --- | --- | --- |
| *M. tuberculosis* | MIC | (12.5% MIC^1^) | reduction | (25% MIC) | reduction | (50% MIC) | reduction |  |
| Vancomycin | 5 | 2.5 | 2 | 1.25 | 4 | 0.625 | 8 |  |
|  |  |  |  |  |  |  |  |  |

All MIC values are represented as mg/L.
Experiments were carried out two independent times in duplicate and representative data are shown.
