# Supplementary material for: 2-aminoimidazoles potentiate ß-lactam antimicrobial activity against Mycobacterium tuberculosis by reducing ß-lactamase secretion and increasing cell envelope permeability
Source: PLoS One. 2017 Jul 27;12(7):e0180925. doi: 10.1371/journal.pone.0180925 (PMC5547695; doi:10.1371/journal.pone.0180925)
Supplement: S2 Table — (DOCX) [file pone.0180925.s008.docx]

|  |  | MIC with SDS | Fold | MIC with SDS | Fold | MIC with SDS | Fold |  |
| --- | --- | --- | --- | --- | --- | --- | --- | --- |
| *M. tuberculosis* | MIC | (12.5% MIC^1^) | reduction | (25% MIC) | reduction | (50% MIC) | reduction |  |
| Carbenicillin | 512 | 512 | 1 | 256 | 2 | 64 | 8 |  |
| Amoxicillin | 512 | 512 | 1 | 256 | 2 | 64 | 8 |  |
| Ceftazidime | 256 | 256 | 1 | 128 | 2 | 32 | 8 |  |
| Meropenem | 8 | 8 | 1 | 4 | 2 | 2 | 4 |  |
| Penicillin V | 512 | 512 | 1 | 256 | 2 | 128 | 4 |  |
|  |  |  |  |  |  |  |  |  |

All MIC values are represented as mg/L.
^1^SDS MIC against *M. tuberculosis* H37Rv was 0.025%.
Experiments were carried out at least two independent times in duplicate and representative data are shown.
